# Supplementary material for: MultiMAP: dimensionality reduction and integration of multimodal data
Source: Genome Biol. 2021 Dec 20;22:346. doi: 10.1186/s13059-021-02565-y (PMC8686224; doi:10.1186/s13059-021-02565-y)
Supplement: Supplementary file 1 — Additional file 1: Figures S1-S9 [file 13059_2021_2565_MOESM1_ESM.docx]

Supplementary Figures


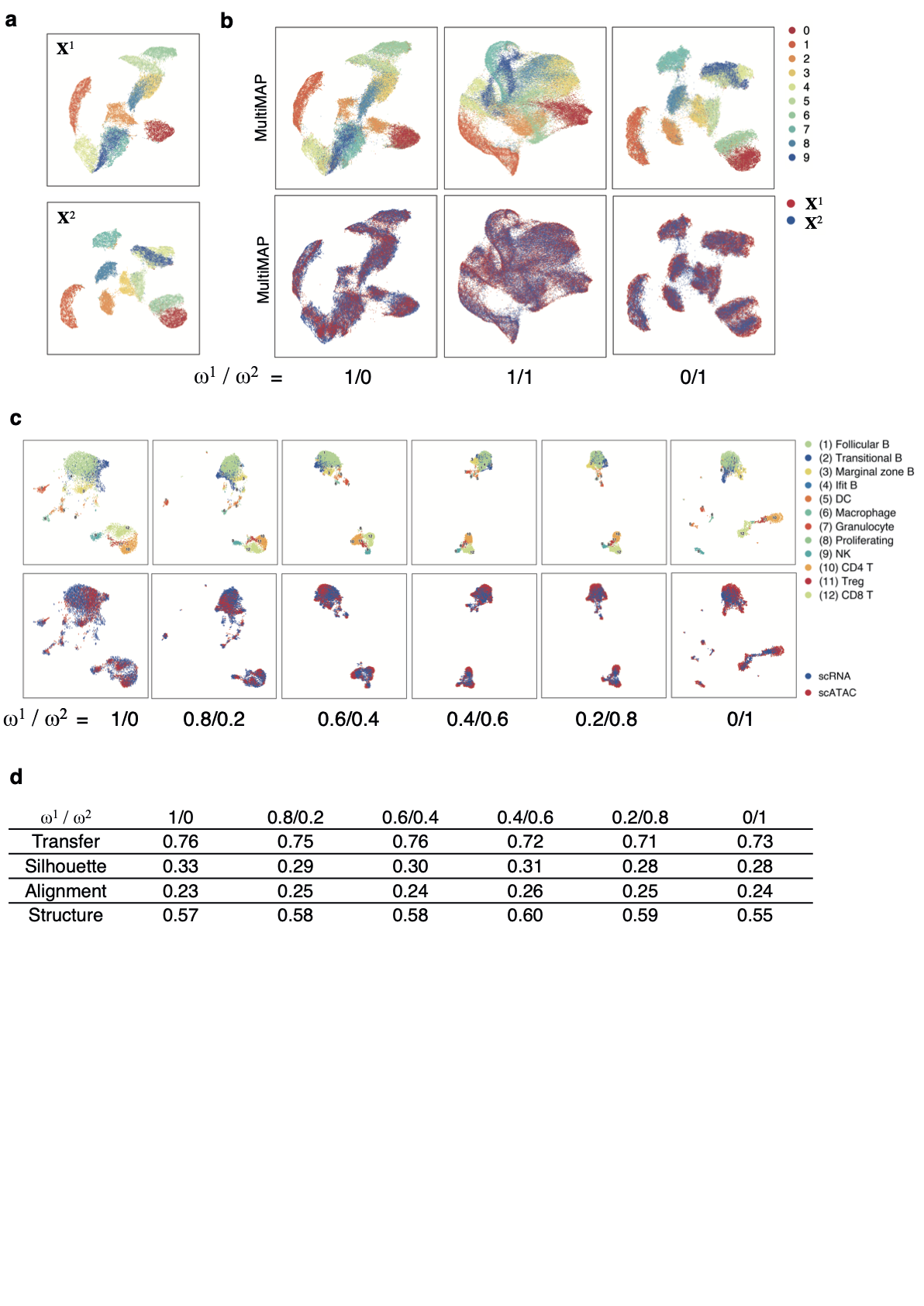


Figure S1. **MultiMAP's weight parameter.** **a.** UMAP projections of the two halves of the MNIST handwritten digit images. **b.** MultiMAP embeddings as the weight parameters are varied. Each color is a different handwritten digit (0-9). When *ω*^1^ is larger than *ω*^2^, the embedding more closely resembles the projection of only **X**^1^; when *ω*^2^ is larger than *ω*^1^, the embedding more closely resembles the projection of only **X**^2^. For different choices of *ω*^v^, the datasets are well integrated in the embedding space. **c.** MultiMAP integration with varied weight parameters of published scATAC-seq[^16^](https://paperpile.com/c/VMMARd/NkGUb) and newly generated scRNA-seq data of the mouse spleen (n=1). **d.** Comparison of the MultiMAP integration of the spleen data as the weight parameter is varied -- in terms of transfer learning accuracy (“Transfer”), separation of cell type clusters as quantified by Silhouette coefficient (“Silhouette”), and preservation of high-dimensional structure as measured by the Pearson correlation between distances in the high- and low-dimensional spaces (“Structure”)


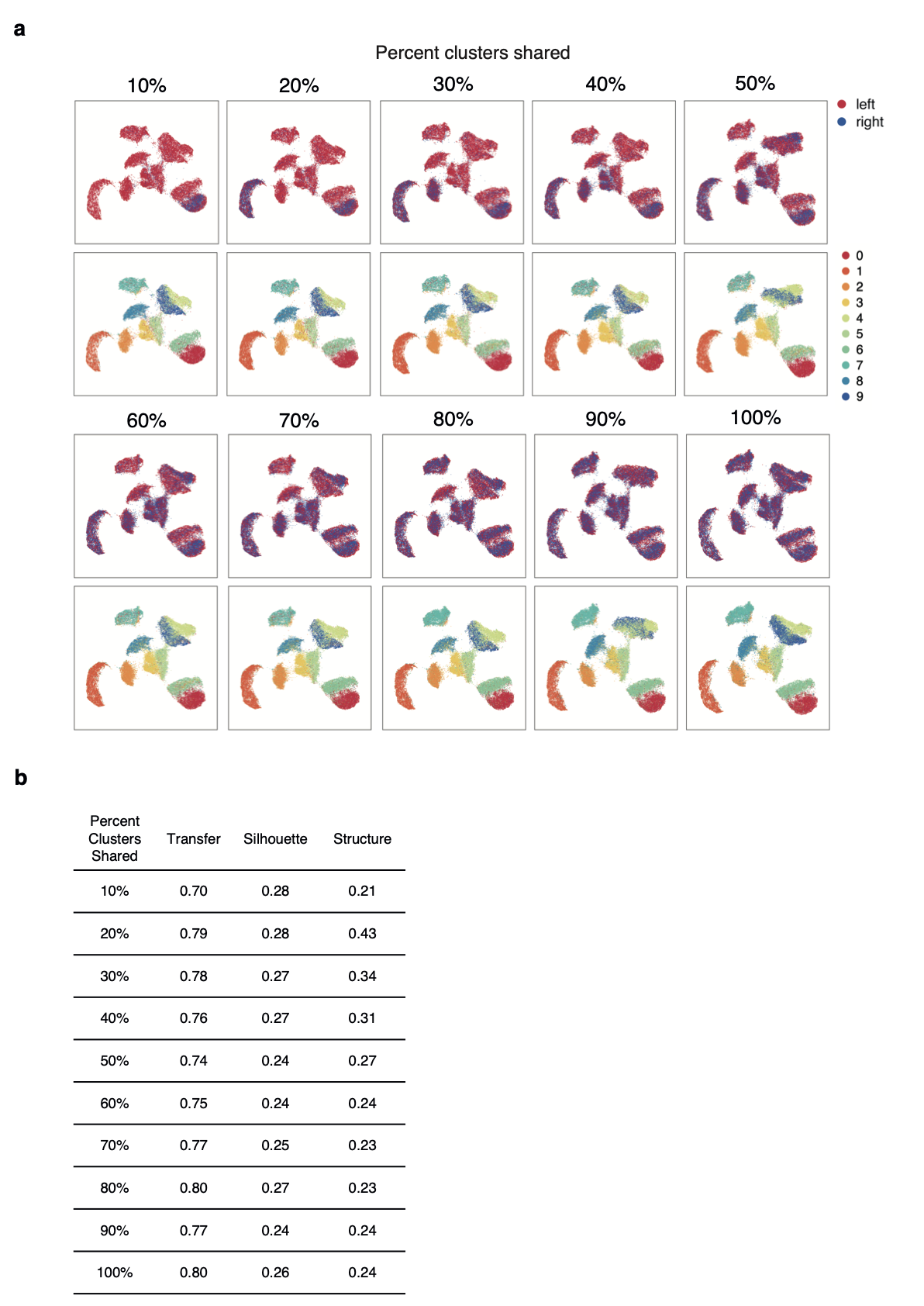


Figure S2. **MultiMAP integration with non-shared clusters. a.** MultiMAP integration of the left and right halves of MNIST handwritten digit images with a 2 pixel wide shared region. Gaussian noise is added to the left half. MultiMAP integration is performed with a varying number of digit clusters removed from the right dataset, so that the integration ranges from one shared cluster (10%) to all clusters shared (100%). **b.** Comparison of the MultiMAP integration of the modified MNIST dataset as the percent of clusters shared is varied -- in terms of transfer learning accuracy (“Transfer”), separation of cell type clusters as quantified by Silhouette coefficient (“Silhouette”), and preservation of high-dimensional structure as measured by the Pearson correlation between distances in the high- and low-dimensional spaces (“Structure”).


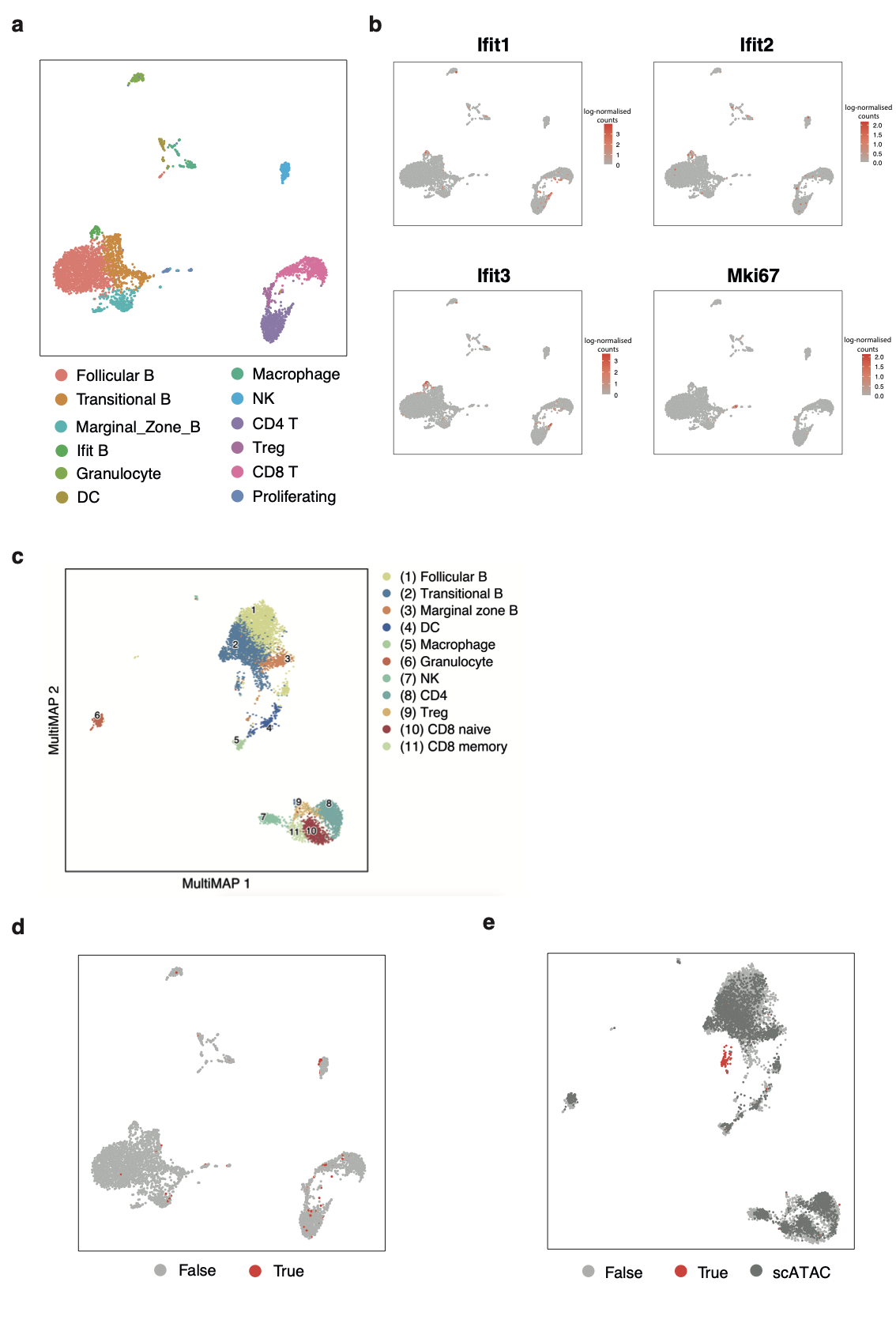


Figure S3. **Mouse spleen scRNA-seq and scATAC-seq data**. **a**. UMAP visualization of the mouse spleen scRNA-seq data (n=1) colored by the identified cell types. **b.** UMAP visualisation of expression levels of Ifit family genes associated with interferon response, upregulated in one specific B cell subpopulation, and the proliferation marker Mki67. **c**. MultiMAP visualization of the integrated scRNA-seq and scATAC-seq mouse spleen data (n=1) colored by the jointly identified clusters. **d, e**. UMAP (d) and MultiMAP (e) visualizations of the mouse spleen data showing cells identified as doublets (labelled “True”) using an independent pipeline (Scrublet). The MultiMAP visualisation leads to these artifactual data points being clustered in one group, highlighting the power of this method to visualise and separate data.


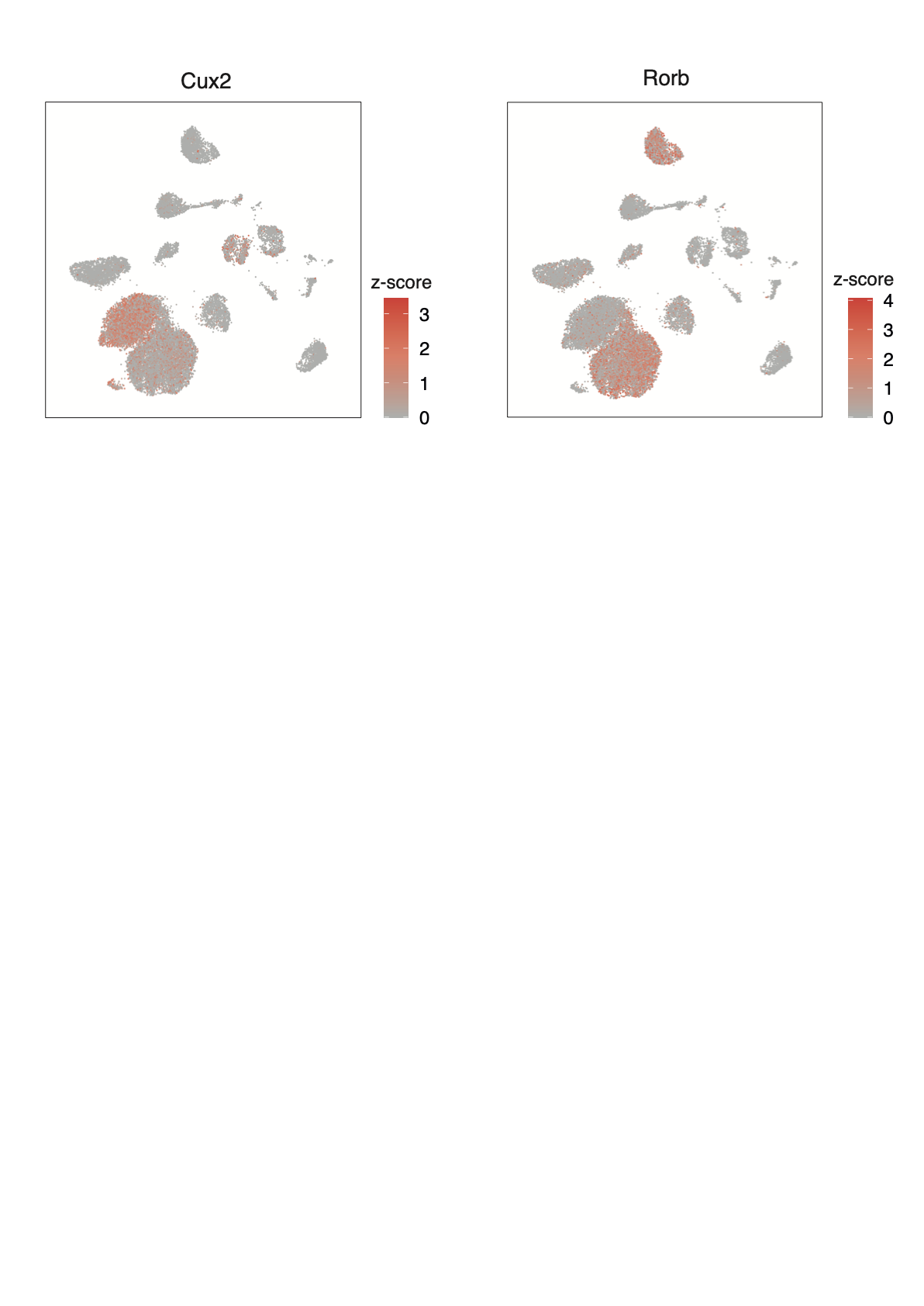


Figure S4. **Marker genes of the L4 cluster identified in the scRNA-seq and STARmap integration.** MultiMAP visualisation of log-transformed gene expression of markers associated with L4 neurons. The MultiMAP integration identified L4 cells in the scRNA-seq data previously annotated as L5 neurons.

**
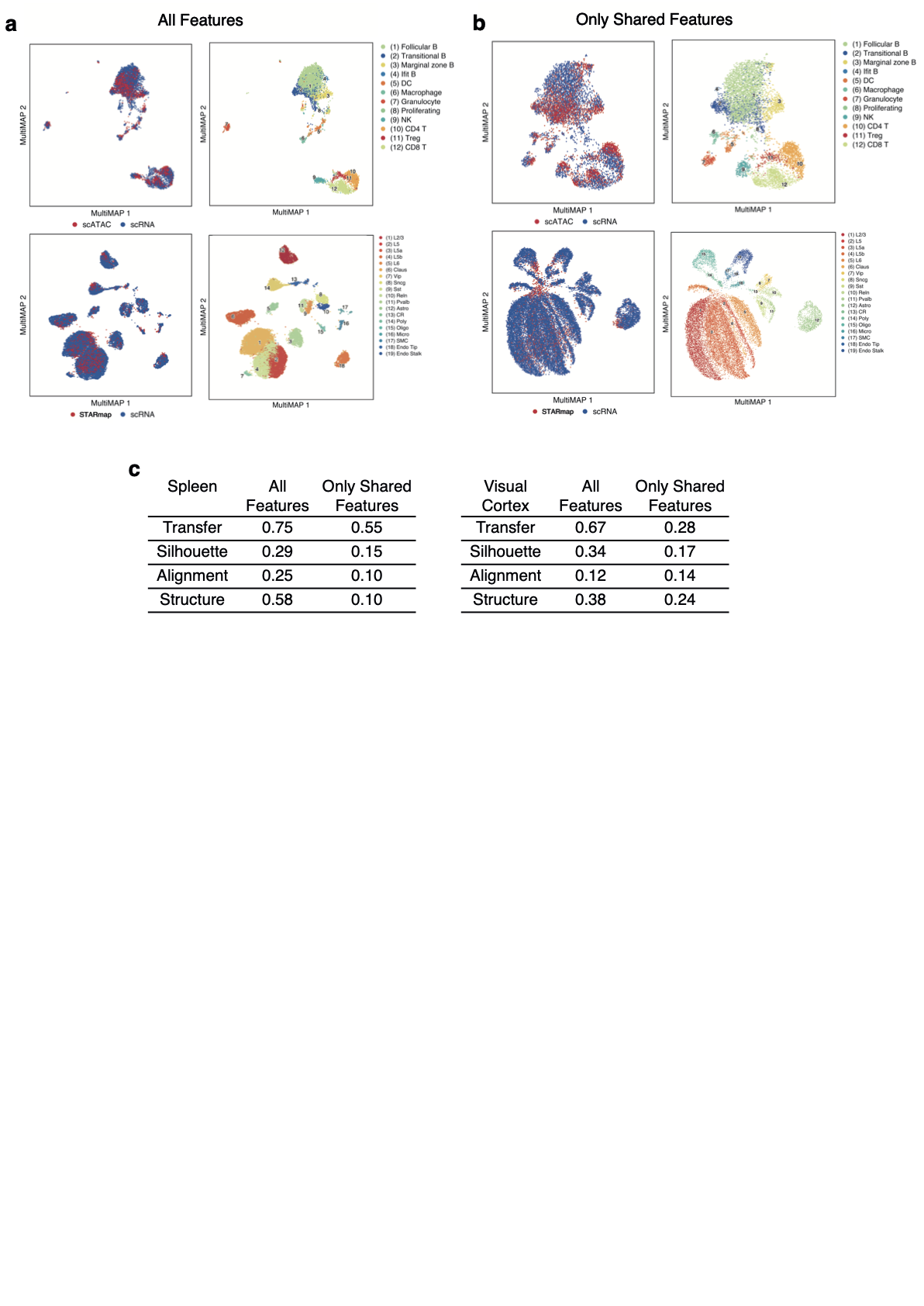
**

Figure S5. **MultiMAP integration with all features vs. only shared features in the spleen scRNA-seq + scATACseq, and visual cortex STARmap + scRNAseq datasets.** **a.** MultiMAP embeddings using all genes present in each dataset (intended use of MultiMAP). **b.** MultiMAP embeddings using only genes shared by all datasets in each integration. **c.** Comparison of the MultiMAP integration with all features vs. only shared features -- in terms of transfer learning accuracy (“Transfer”), separation of cell type clusters as quantified by Silhouette coefficient (“Silhouette”), mixing of different datasets as measured by fraction of nearest neighbours that belong to a different dataset (“Alignment”), and preservation of high-dimensional structure as measured by the Pearson correlation between distances in the high- and low-dimensional spaces (“Structure”).


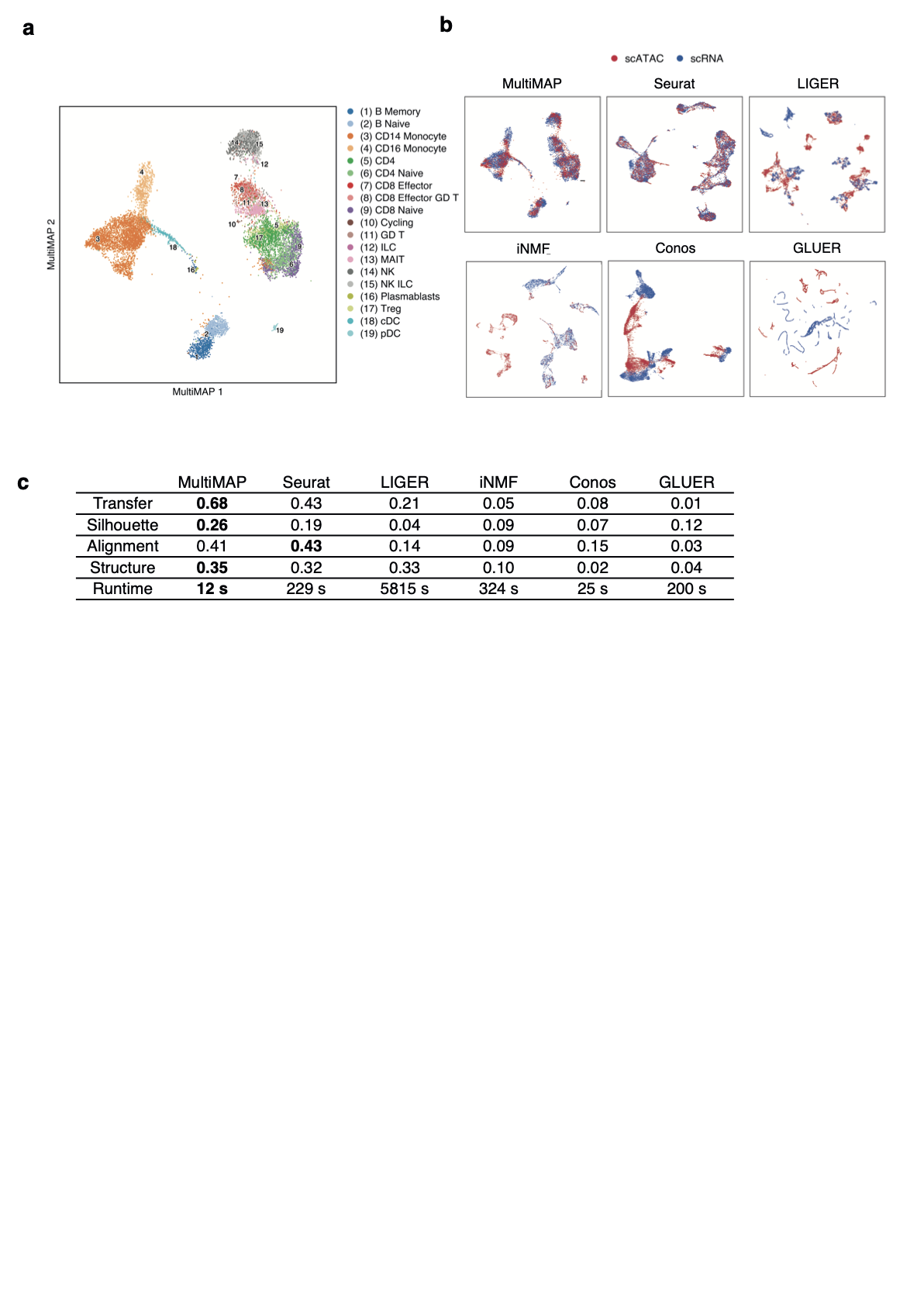


Figure S6. **Benchmarking MultiMAP using paired PBMC data.** **a.** MultiMAP visualization of the Multiome RNA+ATAC PBMCs, colored by independently annotated cell type. **b.** Embeddings produced by alternative integration strategies, colored by omic technology. **c.** Comparison of each method in terms of transfer learning accuracy (“Transfer”), separation of cell type clusters as quantified by Silhouette coefficient (“Silhouette”), mixing of different datasets as measured by fraction of nearest neighbours that belong to a different dataset (“Alignment”), preservation of high-dimensional structure as measured by the Pearson correlation between distances in the high- and low-dimensional spaces (“Structure”), and runtime.


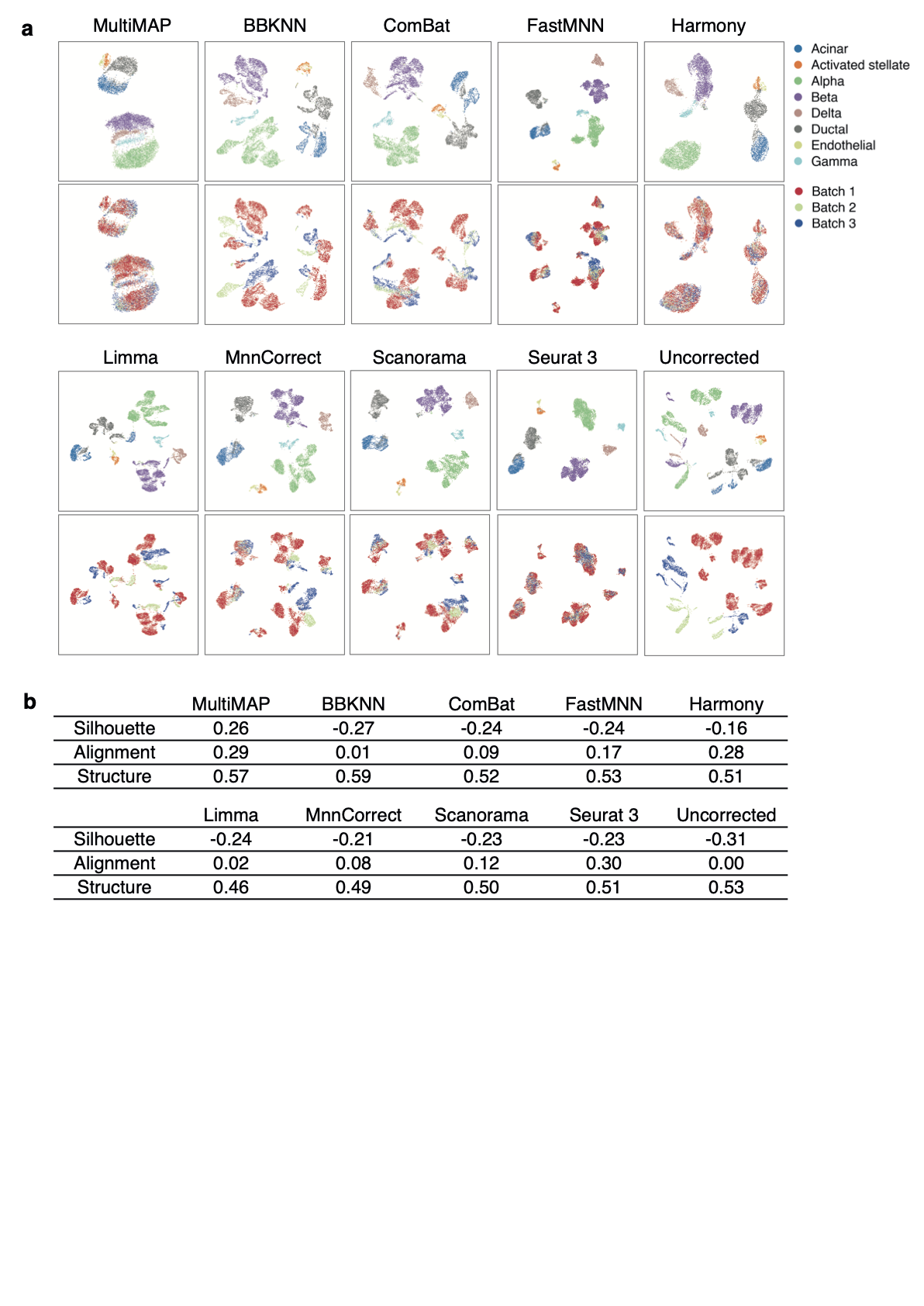


Figure S7. **Benchmarking MultiMAP against batch correction methods.** **a.** Embeddings returned by MultiMAP and batch correction methods on three scRNA-seq pancreas datasets. **b.** Comparison of separation of cell type clusters as quantified by Silhouette coefficient (“Silhouette”), mixing of different datasets as measured by fraction of nearest neighbours that belong to a different dataset (“Alignment”), preservation of high-dimensional structure as measured by the Pearson correlation between distances in the high- and low-dimensional spaces (“Structure”), and runtime.


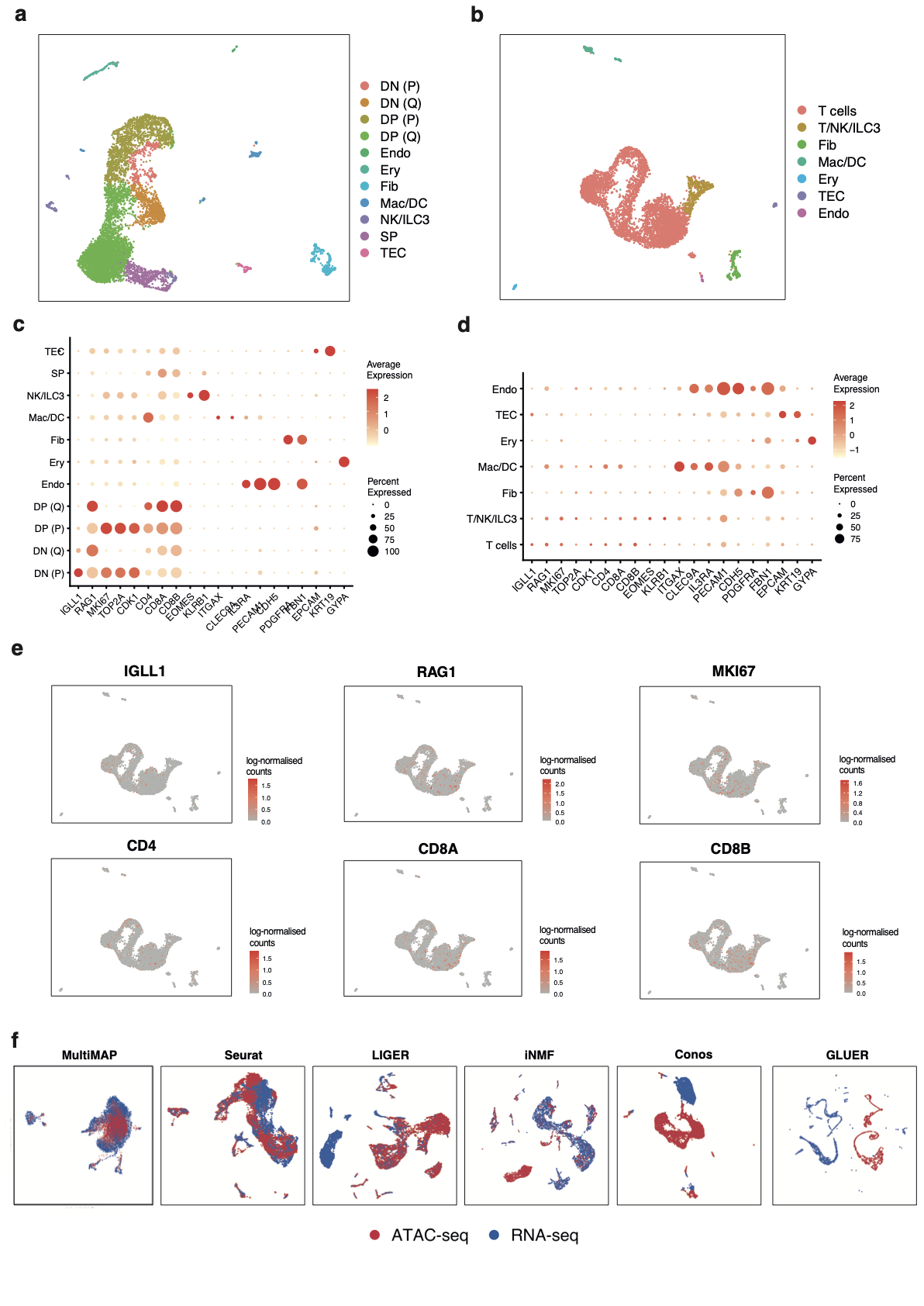


Figure S8. **Fetal thymus scRNA-seq and scATAC-seq data.** **a.** UMAP visualisation of the fetal thymus scRNA-seq data (n=1) colored by identified cell types shows the same cell types as previously published[^32^](https://paperpile.com/c/VMMARd/kAtK8). **b.** UMAP visualisation of the fetal thymus scATAC-seq data (n=1) colored by the identified cell types. **c.** Dot plot showing the z-score of the mean log-transformed expression level of marker genes. **d.** Dot plot showing the z-score of the mean log-transformed gene activity scores of marker genes, showing not very clear separation of T cells clusters in the scATAC-seq data. **e.** UMAP visualisation of log-transformed gene activity scores of markers for specific T cell subpopulations, showing that the scATAC-seq dataset does not separate well the T cell clusters. **f.** Embeddings returned by multi-omic integration methods on the thymus dataset.


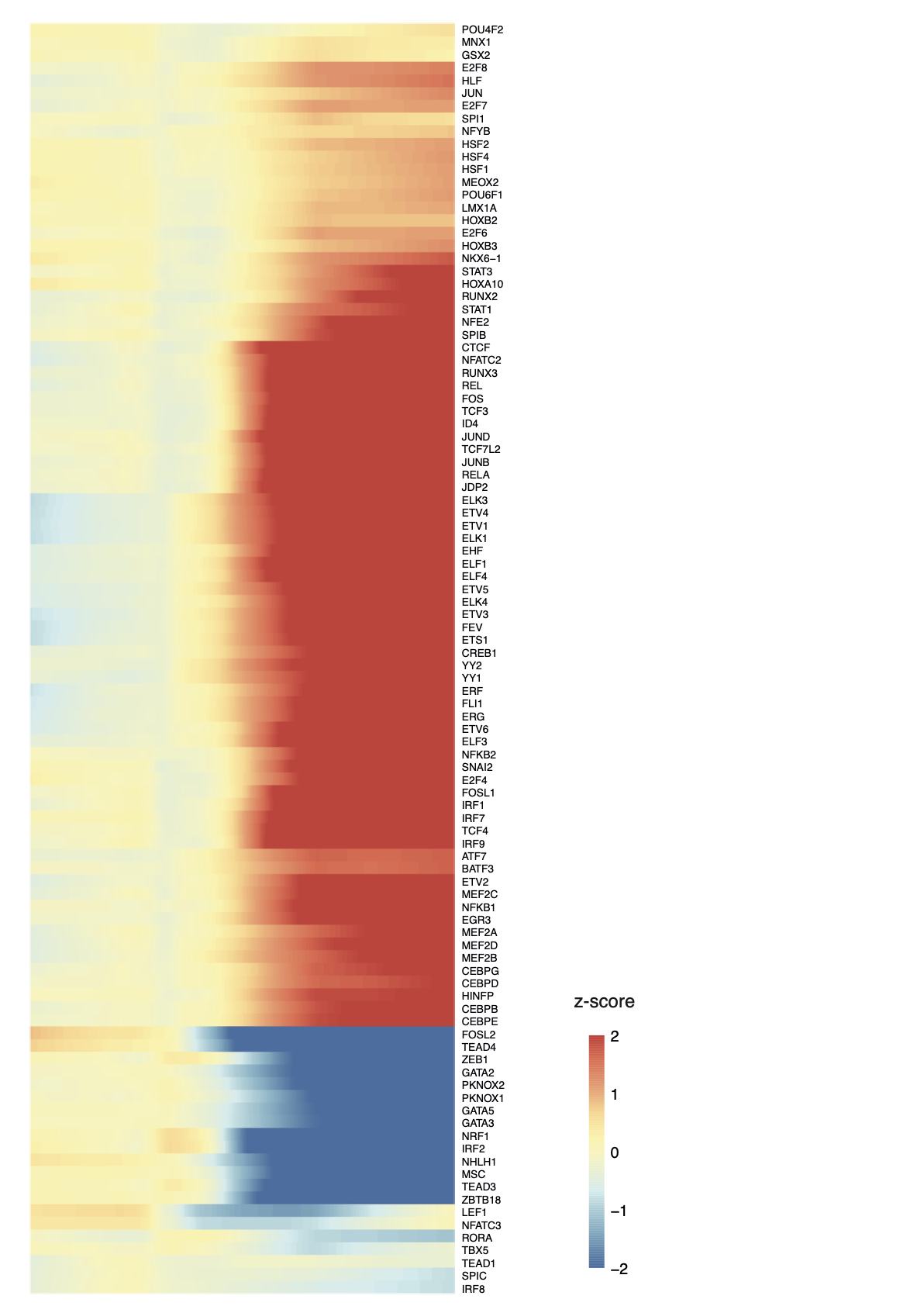


Figure S9. **Chromatin accessibility of transcription factor binding sites.** Smoothed heatmaps of the z-score of motif accessibility of the top 100 most variable transcription factor binding sites over pseudotime. The TF binding sites that varied most in time show changes in accessibility at the transition between the late DN and early DP stage of differentiation.
