# Supplementary material for: MultiMAP: dimensionality reduction and integration of multimodal data
Source: Genome Biol. 2021 Dec 20;22:346. doi: 10.1186/s13059-021-02565-y (PMC8686224; doi:10.1186/s13059-021-02565-y)
Supplement: Supplementary file 2 — Additional file 2. Supplementary Methods [file 13059_2021_2565_MOESM2_ESM.pdf]

Supplementary Methods

Here, we introduce MultiMAP, a new manifold learning approach for the integration and dimensionality reduction of multimodal data. MultiMAP generalizes and extends the mathematical framework of UMAP to the multimodal setting. We emphasize that MultiMAP is a novel algorithm and distinct from UMAP — MultiMAP operates on any number of datasets with different dimensions (UMAP operates on only 1 dataset) and has different graph construction, edge weighting, and optimization as compared to UMAP. Here we review UMAP, introduce a generalized description of multimodal data, and introduce MultiMAP. We also discuss the properties of MultiMAP and study its behavior on synthetic data.

1 UMAP

Uniform manifold approximation and projection (UMAP) is a popular approach for dimensionality reduction due to its fast runtime and striking ability to preserve the structure of data [14,56]. The formulation of UMAP is motivated by ideas from Riemannian geometry, algebraic topology, and fuzzy set theory. We discuss UMAP in preparation for the introduction of MultiMAP. To more smoothly transition to MultiMAP, we modify the notation and presentation found in the original UMAP paper [14]. We also expand on the motivation for some of the steps.

The key steps of UMAP are summarized as follows. UMAP takes the data to be distributed uniformly on a manifold. UMAP estimates geodesic distances between data points on this manifold. These distances are used to construct a fuzzy set representation of the structure of the data on the manifold. A fuzzy set representation of the data is also constructed in a low-dimensional space. UMAP then optimizes the layout of the data in the low-dimensional space to minimize the cross entropy between the two representations.

UMAP takes as input a single high-dimensional dataset $\mathbf{X} = \{\mathbf{x}_1, ..., \mathbf{x}_N \in \mathbb{R}^D\}$ and returns a low-dimensional embedding $\mathbf{Y} = \{\mathbf{y}_1, ..., \mathbf{y}_N \in \mathbb{R}^d\}, d < D$, where $\mathbf{y}_i$ is the projection of $\mathbf{x}_i$. The data is taken to be uniformly distributed on a manifold $\mathcal{M}$ with Riemannian metric g and ambient space $\mathbb{R}^D$. The metric g provides a means to calculate geodesic distances between points on $\mathcal{M}$ given their coordinates in $\mathbb{R}^D$.

UMAP aims to calculate geodesic distances $d_{\mathcal{M}}(p, q)$ between data points $p, q \in \mathcal{M}$. It would be possible to calculate $d_{\mathcal{M}}$ if we knew g, but since we

usually do not, UMAP must resort to other means. Since the data is uniformly distributed on $\mathcal{M}$, any ball on the manifold of fixed radius should contain the same number of data points. Conversely, a ball centered on any point p on the manifold containing k data points should have the same radius. If g is a constant diagonal matrix locally, within the ball, then it follows that we can calculate geodesic distances between p and its k-nearest data points by normalizing euclidean distances with respect to the radius of the ball. This is formalized in Lemma 1, adapted from [14].

Lemma 1 *Let $(\mathcal{M}, g)$ be a Riemannian manifold in an ambient $\mathbb{R}^D$, and let $p \in \mathcal{M}$ be a point. If g is locally constant about p in an open neighbourhood U such that g is a scalar matrix in ambient coordinates, then in a ball $B \subseteq U$ centered at p, the geodesic distance $d_{\mathcal{M}}$ from p to any point $q \in B$ is $\frac{1}{\sigma_p} d_{\mathbb{R}^D}(p, q)$, where σ_p is the radius of the ball in the ambient space and $d_{\mathbb{R}^D}$ is the distance metric in the ambient space.*

Since g is locally a constant diagonal matrix, a point's k-nearest neighbors on $\mathcal{M}$ are the same as its k-nearest neighbors in $\mathbb{R}^D$. We can therefore calculate the geodesic distances

$$d_{\mathcal{M}}(\mathbf{x}_i, \mathbf{x}_j) = \frac{d_{\mathbb{R}^D}(\mathbf{x}_i, \mathbf{x}_j)}{\sigma_i} \text{ for } \mathbf{x}_j \in \mathcal{N}(\mathbf{x}_i\,;\,k),$$

where $\mathcal{N}(\mathbf{x}_i\,;\,k)$ is the set of the k-nearest neighbors of $\mathbf{x}_i$ and σ_i is the radius in $\mathbb{R}^D$ of the ball containing $\mathcal{N}(\mathbf{x}_i\,;\,k)$. A robust way to estimate σ_i is discussed later in this section.

UMAP uses $d_{\mathcal{M}}$ to construct a representation of the data distribution on $\mathcal{M}$. This representation takes the form of a fuzzy set (A, μ) defined by a reference set A and a membership strength function $\mu\colon A \to [0, 1]$. UMAP constructs a *fuzzy simplicial set*, with A being the set of edges (1-simplices) that connect neighboring points. This fuzzy simplicial set can also be viewed as a weighted k-nearest neighbor graph (k-NNG), with nodes $\{1, ..., N\}$, edges A, and edge weights μ. Concretely,

$$\begin{aligned}
A &= \{\{i, j\} \mid i = 1, ..., N, \mathbf{x}_j \in \mathcal{N}(\mathbf{x}_i\,;\,k)\}. \\
\mu(\{i, j\}) &= \mu_{i|j} + \mu_{j|i} - \mu_{i|j}\mu_{j|i} \\
\mu_{i|j} &= \exp(-\max(0, d_{\mathcal{M}}(\mathbf{x}_i, \mathbf{x}_j) - \rho_i)) \\
\mu_{i|j} &= \begin{cases} \exp(-\max(0, d_{\mathcal{M}}(\mathbf{x}_i, \mathbf{x}_j) - \rho_i)) & \text{if } \{i, j\} \in A, \\ 0 & \text{otherwise} \end{cases} \\
\rho_i &= \min_j d_{\mathcal{M}}(\mathbf{x}_i, \mathbf{x}_j).
\end{aligned}$$

The value of μ is larger when points are closer together on the manifold. The particular form of μ is motivated by results from algebraic topology and category theory [1,57,58]. A number of packages can be used to efficiently compute approximate nearest neighbors in $\mathbb{R}^D$.

To finish constructing the fuzzy simplicial set, σ_i still needs to be estimated. Since the data is uniformly distributed on $\mathcal{M}$, the geodesic distances between neighboring data points are i.i.d. and so we expect $\sum_j \mu_{i|j}$ to be the same for each i. Therefore, σ_i is taken to satisfy the relation

$$\sum_{j \in \{l \mid \{i,l\} \in A\}} \exp\left[\frac{-\max\left(0, d_{\mathbb{R}^D}(\mathbf{x}_i, \mathbf{x}_j) - \min_{\{i,l\} \in A} d_{\mathbb{R}^D}(\mathbf{x}_i, \mathbf{x}_l)\right)}{\sigma_i}\right] = c,$$

where c is some constant. UMAP sets c to $\log_2(k)$ based on empirical results. Binary search is used to estimate the values of σ_i that satisfy this relation.

UMAP also constructs a fuzzy set (A, ν) representation of the data in the low-dimensional space $\mathbb{R}^d$. Ultimately, UMAP optimizes the layout of the data so that (A, ν) resembles (A, μ). We are not interested in creating a fuzzy set that accurately captures the manifold structure of $\mathbb{R}^d$. So a simpler form of the membership function is used that encourages points close on $\mathcal{M}$ to also be close in the low-dimensional space while keeping ν in the range [0,1]. The membership function in the low-dimensional spaces is defined as

$$\nu(\{i,j\}) = (1 + a\|\mathbf{y}_i - \mathbf{y}_j\|_2^{2b})^{-1},$$

where a and b are user-defined positive values.

To quantify the difference between the fuzzy set of the data on the manifold and the fuzzy set in the low-dimensional space, UMAP uses fuzzy set cross entropy [59]. The cross entropy of (A, ν) relative to (A, μ) is given by

$$C((A,\mu),(A,\nu)) = \sum_{a \in A} \mu(a) \log\left(\frac{\mu(a)}{\nu(a)}\right) + (1 - \mu(a)) \log\left(\frac{1 - \mu(a)}{1 - \nu(a)}\right).$$

UMAP initializes $\mathbf{Y}$ to the spectral layout of the k-NNG, and then uses stochastic gradient descent to minimize C with respect to $\mathbf{Y}$. This optimization scales linearly with the number of datapoints, and so is quite efficient. The optimized $\mathbf{Y}$ is returned as the low-dimensional projection of the data.

2 Generalized Multimodal Setting

Here, we introduce a generalized description of multimodal data, which will be the setting of MultiMAP. In this setting, we have multiple datasets $\{\mathbf{X}^v \mid v = 1, ..., V\}$. Each is a set of data points, $\mathbf{X}^v = \{\mathbf{x}_1^v, ..., \mathbf{x}_{N^v}^v \in \mathbb{R}^{D^v}\}$. Different datasets can be measurements made with different technologies, under different conditions, and/or of different systems. To keep this setting as general as possible, we allow each dataset to have different dimensionality. Throughout this work, lower indices are for data points and upper indices are for datasets. The total number of data points is $N = \sum_v N^v$.

We also assume we can calculate distances between data points in different datasets. To further preserve the generality of the setting, we take these

distances to be defined between select pairs of datasets, for a select number of points, in a potentially limited feature space. The existence of only some distances is what characterizes this setting as multimodal. If distances were defined between every point using the the same features, all data would reside in a shared feature space. Concretely, distances can be calculated between members of $\mathbf{X}^{uv} = \{\tilde{\mathbf{x}}_i^u, \tilde{\mathbf{x}}_j^v, ... \in \mathbb{R}^{\tilde{D}^{uv}} \mid i \in I^{uv} \subseteq \{1, ..., N^u\},\ j \in J^{uv} \subseteq \{1, ..., N^v\}\}$ for $(u, v) \in S \subseteq \{(u, v) \mid u, v = 1, ..., V\}$. Distances can be calculated based on features, labels, or points shared between datasets. We take $\mathbf{X}^{vv} = \mathbf{X}^v$ and $I^{vv} = J^{vv} = \{1, ..., N^v\}$.

So in summary, in the generalized multimodal setting, we are given $\{\mathbf{X}^{uv}\}$, $\{I^{uv}\}$, $\{J^{uv}\}$, and S. Alternatively, instead of $\{\mathbf{X}^{uv}\}$, we can be given directly the distances D_{ij}^{uv} between $\tilde{\mathbf{x}}_i^u$ and $\tilde{\mathbf{x}}_j^v$ in the space $\mathbb{R}^{\tilde{D}^{uv}}$.

This multimodal setting is "generalized" because we do not assume the data all reside in the same feature space, nor that the datasets have the same dimensionality, nor that distances are defined between every pair of data points. As we discuss later, many approaches for multimodal integration require that every data point reside in exactly the same feature space, a setting which makes all of these assumptions. In contrast, approaches in the generalized multimodal setting can potentially leverage features not present in all datasets, define distances between certain data using side information such as class labels, and eliminate distances between certain data points if, for example, they are noisy or uncertain.

3 Foundations of MultiMAP

Here we introduce Multimodal Manifold Approximation and Projection (MultiMAP). MultiMAP is an approach to dimensionality reduction which projects multimodal data into a single low-dimensional embedding. Whereas traditional dimensionality reduction methods project a single dataset, MultiMAP operates on multiple datasets simultaneously to identify a projection that is suitable for all of the data. MultiMAP builds upon the framework of UMAP to extend it to the generalized multimodal setting. In doing so, MultiMAP preserves and extends the mathematical motivation and computational efficiency of UMAP. We emphasize that MultiMAP is a novel algorithm and distinct from UMAP — MultiMAP operates on any number of datasets with different dimensions (UMAP operates on only 1 dataset) and has different graph construction, edge weighting, and optimization as compared to UMAP.

A principle hypothesis of MultiMAP is that if different data modalities characterize the same underlying system, the data from each modality should be uniformly distributed on a single latent manifold. We refer to this idea as the *multimodal manifold hypothesis*. This manifold is single landscape on which data from different modalities reside in an integrated and unified manner. In general, the manifold is an abstract object that does not exist in the coordinate system of any particular dataset. In the context of single-cell measurements, this manifold can be thought to be the landscape of cell states, with distinct

regions corresponding to cell types and continuous regions corresponding to transitions between cell types. MultiMAP aims to recover the structure of the data distribution on the manifold and project it into a low-dimensional space.

A second principle hypothesis of MultiMAP is that if data points from different modalities are close together on the latent manifold, they will also be close in the coordinate systems of each of the datasets. We refer to this idea as the *invariance of similarity.* This can be quite a robust assumption: measurement differences often produce global shifts that keep neighboring points in the same vicinity. For example, affine transformations, smooth nonlinear distortions, and random noise generally keep close points together. In the context of single-cell measurements, if cells have a similar underlying biological state (belong to the same type or are at a similar point in a dynamic trajectory) they likely exhibit similar transcriptomes, epigenomes, proteomes, and other characteristics. Visualizations showing that cells of the same type cluster together for a variety of omics, batches, individuals, and species confirm that cell similarity is preserved across modality. MultiMAP leverages the invariance of similarity to recover information about the manifold from the multimodal data.

The key steps of MultiMAP are depicted in Figure 1 and summarized as follows. MultiMAP takes the multimodal data to be distributed uniformly on a manifold. MultiMAP estimates geodesic distances on this manifold between data points. These distances are used to construct a fuzzy set representation of the structure of the data on the manifold. A fuzzy set representation of the data is also constructed in a low-dimensional space. MultiMAP then optimizes the layout of the data in the low-dimensional space to minimize a weighted cross entropy between the two representations. While at a high-level these steps mirror those of UMAP, the mathematical formulation and computation of each step differs to account for the multimodal setting.

MultiMAP operates in the generalized multimodal setting and takes as input $\{\mathbf{X}^{uv}\}$ or $\{D_{ij}^{uv}\}$, in addition to $\{I^{uv}\}$, $\{J^{uv}\}$, and S, as described in the previous section. MultiMAP returns an embedding $\{\mathbf{Y}^v = \{\mathbf{y}_1^v, ..., \mathbf{y}_{N^v}^v \in \mathbb{R}^d\}\}$ of all of the data into a single low-dimensional space, where $\mathbf{y}_i^v$ is the projection of $\mathbf{x}_i^v$. MultiMAP can also return a graph, which we call the MultiGraph, consisting of all of the data integrated into a single graph structure. We describe the MultiGraph in more detail later in this section.

Motivated by the multimodal manifold hypothesis, MultiMAP takes the data to be uniformly distributed on a manifold $\mathcal{M}$ with Riemannian metric g. In the generalized multimodal setting, different data points on $\mathcal{M}$ exist also in different feature spaces. We therefore allow $\mathcal{M}$ to have multiple ambient spaces. Each ambient space $\mathbb{X}^{uv}$ is the feature space of dataset $\mathbf{X}^{uv}$. In general, g is different with respect to each of these ambient spaces, so we denote g^{uv} to be the metric in the coordinates of $\mathbb{X}^{uv}$. The metric g^{uv} gives a way to calculate geodesic distances between points on $\mathcal{M}$ given the coordinates of the points in $\mathbb{X}^{uv}$.

MultiMAP seeks to estimate geodesic distances between points on $\mathcal{M}$. This is challenging since we usually do not know g^{uv}. But we can make headway if we can say something further about g^{uv}. Motivated by the invariance of similarity,

we take g^{uv} to have a form that results in points close on $\mathcal{M}$ also being close in the ambient space $\mathbb{X}^{uv}$. Specifically, we take g^{uv} to be a constant diagonal matrix within a ball $B(p)$ centered at point $p \in \mathcal{M}$ that contains the k nearest data points to p. We do allow g^{uv} to be different for each u, v, and p. If g^{uv} takes this form, data points are nearest neighbors in $\mathbb{X}^{uv}$ if and only if they are nearest neighbors on $\mathcal{M}$, preserving a sense of similarity between p and its neighbors. This is formalized in Lemma 2, which is proven at the end of this section.

Lemma 2 *Let $(\mathcal{M}, g^{uv})$ be a Riemannian manifold with an ambient space $\mathbb{X}^{uv}$, $p \in \mathcal{M}$ be a point, and P be a finite set of points on $\mathcal{M}$. Let $\mathcal{N}_{\mathcal{M}}(p,k) \subseteq P$ be the set of k points that are nearest to p on $\mathcal{M}$, and $\mathcal{N}^{uv}(p,k) \subseteq P$ be the set of k point that are the nearest to p in $\mathbb{X}^{uv}$. If within a ball B centered at p and containing $\mathcal{N}_{\mathcal{M}}(p,k)$, g^{uv} is locally constant such that it is a scalar matrix in ambient coordinates, then $\mathcal{N}_{\mathcal{M}}(p,k) = \mathcal{N}^{uv}(p,k)$.*

Since g^{uv} is a constant diagonal matrix within $B(p)$, it follows that we can calculate geodesic distances between p and its k-nearest data points by normalizing distances in $\mathbb{X}^{uv}$ with respect to the radius of the ball. This is formalized in Lemma 3, which adapts the notation of Lemma 1 to the multimodal setting.

Lemma 3 *Let $(\mathcal{M}, g^{uv})$ be a Riemannian manifold with an ambient space $\mathbb{X}^{uv}$, and let $p \in \mathcal{M}$ be a point. If g^{uv} is locally constant about p in an open neighbourhood U such that g^{uv} is a scalar matrix in the coordinates of $\mathbb{X}^{uv}$, then in a ball $B \subseteq U$ centered at p, the geodesic distance $d_{\mathcal{M}}$ from p to any point $q \in B$ is $\frac{1}{\sigma_p^{uv}} d_{\mathbb{X}^{uv}}(p,q)$, where σ_p^{uv} is the radius of the ball in $\mathbb{X}^{uv}$ and $d_{\mathbb{X}^{uv}}$ is the distance metric in $\mathbb{X}^{uv}$.*

This allows calculation of the geodesic distances

$$d_{\mathcal{M}}(\mathbf{x}_i^u, \mathbf{x}_j^v) = \frac{D_{ij}^{uv}}{\sigma_i^{uv}}, \text{ for } \mathbf{x}_j^v \in B(\mathbf{x}_i^u), (u,v) \in S, i \in I^{uv}, j \in J^{uv},$$

where σ_i^{uv} is the radius of $B(\mathbf{x}_i^u)$ in the ambient space $\mathbb{X}^{uv}$. The restricted values of u, v, i, and j reflect the fact that we only know some D_{ij}^{uv} in the generalized multimodal setting.

To calculate $d_{\mathcal{M}}$, we need an approach to determine if $\mathbf{x}_j^v \in B(\mathbf{x}_i^u)$. Per the multimodal manifold hypothesis, since all of the data are uniformly distributed on $\mathcal{M}$, we expect $B(x_i^u)$ to contain kN^v/N data points from $\mathbf{X}^v$. We also expect $B(\mathbf{x}_i^u)$ to contain $k|J^{uv}|/N$ data points $\mathbf{x}_j^v$, $j \in J^{uv}$, as long as J^{uv} can be approximated as a random sample of $\{1, ..., N^v\}$. By Lemma 2, these $\mathbf{x}_j^v$ are nearest neighbors of $\mathbf{x}_i^u$ in $\mathbb{X}^{uv}$. Since we have pairwise distances between all data in $\mathbb{X}^{uv}$, we can calculate these nearest neighbors, giving us points in $B(\mathbf{x}_i^u)$. We can therefore estimate the geodesic distances

$$d_{\mathcal{M}}(\mathbf{x}_i^u, \mathbf{x}_j^v) = \frac{D_{ij}^{uv}}{\sigma_i^{uv}}, \text{ for } \mathbf{x}_j^v \in \mathcal{N}^{uv}\left(\mathbf{x}_i^u; \left\lfloor \frac{k|J^{uv}|}{N} \right\rceil\right), (u,v) \in S, i \in I^{uv}, j \in J^{uv},$$

where $\mathcal{N}^{uv}(\mathbf{x}_i^u\,;\,K)$ is the set of K points $\mathbf{x}_i^v \in \mathbf{X}^{uv}$ that are closest to $\mathbf{x}_i^u$ in the space $\mathbb{X}^{uv}$. The value of K must be an integer, so $k|J^{uv}|/N$ is rounded. A robust way to estimate σ_i^{uv} is discussed later in this section.

MultiMAP uses $d_{\mathcal{M}}$ to construct a representation of the data distribution on $\mathcal{M}$. Like UMAP, this representation takes the form of a fuzzy set (A, μ), defined by a reference set A and a membership strength function $\mu\colon A \to [0, 1]$. MultiMAP constructs a fuzzy simplicial set, A being the set of edges (1-simplices) that connect neighboring points, and μ taking on a larger value if the points are closer together on the manifold. In the generalized multimodal setting, we must construct (A, μ) differently from UMAP. Since we cannot define geodesic distances between some pairs of points, we must leave out edges connecting these pairs. The fuzzy simplicial set is defined as

$$
\begin{aligned}
A &= \left\{ a_{ij}^{uv} = \{\mathbf{x}_i^u, \mathbf{x}_j^v\} \,\middle|\, u, v = 1, ..., V, i \in I^{uv}, j \in J^{uv}, \mathbf{x}_j^v \in \mathcal{N}^{uv}\left(\mathbf{x}_i^u\,;\, \left\lfloor \frac{k|J^{uv}|}{N} \right\rceil \right) \right\} \\
\mu(a_{ij}^{uv}) &= \mu_{i|j}^{uv} - \mu_{j|i}^{uv} + \mu_{i|j}^{uv}\mu_{j|i}^{uv} \\
\mu_{i|j}^{uv} &= \begin{cases} \exp(-\max(0, d_{\mathcal{M}}(\mathbf{x}_i^u, \mathbf{x}_j^v) - \rho_i^u)) & \text{if } a_{ij}^{uv} \in A, \\ 0 & \text{otherwise} \end{cases} \\
\rho_i^u &= \min_{\mathbf{x}_j^v} d_{\mathcal{M}}(\mathbf{x}_i^u, \mathbf{x}_j^v).
\end{aligned}
$$

The form of μ is the same as that of UMAP and is motivated in [14]. The fuzzy simplicial set can also be viewed as graph with nodes $\{\mathbf{x}_i^v\}$, edges A, and edge weights μ. We call this the MultiGraph, as it integrates multimodal data into a single graph structure. The MultiGraph connects data points if they share a neighborhood on $\mathcal{M}$. The MultiGraph is generally not a k-nearest neighbor graph because its nodes can differ in degree. As we discuss later, the MultiGraph is itself useful for integrated analyses.

To finish constructing the fuzzy simplicial set, we still need to estimate σ_i^{uv}. Per the multimodal manifold hypothesis, since the data is uniformly distributed on $\mathcal{M}$, we expect $\sum_{v,j} \mu_{i|j}^{uv}$ to be the same for each $\mathbf{x}_i^v$. Say this sum is equal to the constant c. The multimodal manifold hypothesis further states that every individual dataset is uniform on $\mathcal{M}$. Thus if we fix v, we expect $\sum_j \mu_{i|j}^{uv}$ to still be the same for each $\mathbf{x}_i^u$. Since the sum now has $|J^{uv}|$ terms instead of N, it should equal $c|J^{uv}|/N$, as long as J^{uv} can be approximated as a random sample of $\{1, ..., N^V\}$. Further, if k is reasonably large, $\rho_i^u = \min_{\mathbf{x}_j^v} d_{\mathcal{M}}(\mathbf{x}_i^u, \mathbf{x}_j^v)$ will be about the same for any choice of v. We can therefore take σ_i^{uv} to satisfy the relation

$$
\sum_{j \in \{l \,|\, a_{il}^{uv} \in A\}} \exp\left[\frac{-\max\left(0, D_{ij}^{uv} - \min\limits_{a_{il}^{uv} \in A} D_{il}^{uv}\right)}{\sigma_i^{uv}} \right] = c\left(\frac{|J^{uv}|}{N}\right). \tag{1}
$$

We set c to $\log_2(k)$ to be consistent with UMAP. Binary search is used to estimate the values of σ_i^{uv} that satisfy this relation.

MultiMAP proceeds to construct a fuzzy set (A, ν) representation of the data in the low-dimensional space $\mathbb{R}^d$. Ultimately, MultiMAP optimizes the layout of the data so that (A, ν) resembles (A, μ). We are not interested in creating a fuzzy set that accurately captures the manifold structure of $\mathbb{R}^d$. So we use a simpler form of the membership function that encourages points close on $\mathcal{M}$ to also be close in the low-dimensional space, while keeping ν in the range [0,1] as required by the definition of fuzzy sets. As in UMAP, the membership function in the low-dimensional spaces is defined as

$$\nu(a_{ij}^{uv}) = (1 + a\|\mathbf{y}_i^u - \mathbf{y}_j^v\|_2^{2b})^{-1},$$

where a and b are user-defined positive values.

To quantify the difference between the fuzzy set of the data on the manifold and the fuzzy set in the low-dimensional space, like UMAP, MultiMAP uses fuzzy set cross entropy. The weighted cross entropy of (A, ν) relative to (A, μ) is given by

$$C((A, \mu), (A, \nu)) = \sum_{a_{ij}^{uv} \in A} \mu(a_{ij}^{uv}) \log\left(\frac{\mu(a_{ij}^{uv})}{\nu(a_{ij}^{uv})}\right) + (1 - \mu(a_{ij}^{uv})) \log\left(\frac{1 - \mu(a_{ij}^{uv})}{1 - \nu(a_{ij}^{uv})}\right).$$

MultiMAP initializes $\{\mathbf{Y}^v\}$ to the spectral layout of the MultiGraph, and then uses stochastic gradient descent to optimize $\{\mathbf{Y}^v\}$ to minimize the cross entropy. The optimized $\{\mathbf{Y}^v\}$ is returned as the low-dimensional projection of the data.

Unlike UMAP, MultiMAP uses a weighted optimization scheme. The contribution of a point to the low-dimensional layout is weighted by the dataset it originates from. The gradient updates, with learning rate α, are given by

$$\mathbf{y}_i^u \leftarrow \mathbf{y}_i^u + \alpha \sum_{a_{ij}^{uv} \in A} \frac{V\omega^v}{\sum_v \omega^v} \frac{\partial}{\partial \mathbf{y}_i^u} \left[\mu(a_{ij}^{uv}) \log\left(\frac{\mu(a_{ij}^{uv})}{\nu(a_{ij}^{uv})}\right) + (1 - \mu(a_{ij}^{uv})) \log\left(\frac{1 - \mu(a_{ij}^{uv})}{1 - \nu(a_{ij}^{uv})}\right) \right].$$

The weight $\omega^v \in \mathbb{R}$ controls the influence of dataset $\mathbf{X}^v$ on the final embedding. When ω^v has a larger relative value, the layout of all of the data will depend more strongly on $\mathbf{X}^v$. This is especially useful when one dataset is known to be of higher/lower quality, in which case its ω^v can be set larger/smaller, respectively. In the case of two datasets $\{\mathbf{X}^1, \mathbf{X}^2\}$, setting ω^1=0 and ω^2=1 pulls the layout of the first dataset to that of the second dataset, without the first influencing the layout of the second. This can be useful for "aligning" a query dataset $\mathbf{X}^1$ of unknown content or quality to a reference dataset $\mathbf{X}^2$. The default value of all ω^v is 1, which equally weights the influence of all datasets. When all w^v are the same, the optimization of MultiMAP is equivalent to that of UMAP.

To deal with the unique challenges of the multimodal setting, MultiMAP is different from UMAP in several regards. MultiMAP differs from UMAP in its estimation of geodesic distances, construction of the fuzzy simplicial set, and

use of a weighted cross entropy. The motivation of MultiMAP also incorporates new ideas including the multimodal manifold hypothesis and the invariance of similarity. These distinctions allow MultiMAP to deal with datasets of different dimensions and feature spaces, unknown distances between data in different datasets, and datasets of varying quality. MultiMAP can be seen as a generalization of UMAP to the multimodal setting, exactly reducing to UMAP when $V=1$.

Proof of Lemma 2

Lemma 2 *Let $(\mathcal{M}, g^{uv})$ be a Riemannian manifold with an ambient space $\mathbb{X}^{uv}$, $p \in \mathcal{M}$ be a point, and P be a finite set of points on $\mathcal{M}$. Let $\mathcal{N}_{\mathcal{M}}(p,k) \subseteq P$ be the set of k points that are nearest to p on $\mathcal{M}$, and $\mathcal{N}^{uv}(p,k) \subseteq P$ be the set of k point that are the nearest to p in $\mathbb{X}^{uv}$. If within a ball B centered at p and containing $\mathcal{N}_{\mathcal{M}}(p,k)$, g^{uv} is locally constant such that it is a scalar matrix in ambient coordinates, then $\mathcal{N}_{\mathcal{M}}(p,k) = \mathcal{N}^{uv}(p,k)$.*

We show that each point in $\mathcal{N}_{\mathcal{M}}(p,k)$ is in $\mathcal{N}^{uv}(p,k)$ and each point in $\mathcal{N}^{uv}(p,k)$ is in $\mathcal{N}_{\mathcal{M}}(p,k)$. For convenience we let $\mathcal{N}_{\mathcal{M}} = \mathcal{N}_{\mathcal{M}}(p,k)$ and $\mathcal{N}^{uv} = \mathcal{N}^{uv}(p,k)$.

Consider a datapoint P such that $P \in \mathcal{N}_{\mathcal{M}}$ and $P \notin \mathcal{N}^{uv}$. There must be at least k points $\tilde{p}_i \neq P$ such that $d_{\mathbb{X}^{uv}}(p,\tilde{p}_i) < d_{\mathbb{X}^{uv}}(p,P)$. Because $\tilde{p}_i$ is closer than P to p in $\mathbb{X}^{uv}$, and $P \in B$, it must be the case that $\tilde{p}_i \in B$. Since g^{uv} is a constant diagonal matrix at every point within B, by Lemma 3, $d_{\mathcal{M}}(p,P) = d_{\mathbb{X}^{uv}}(p,P)/\sigma_i^{uv}$ and $d_{\mathcal{M}}(p,\tilde{p}_i) = d_{\mathbb{X}^{uv}}(p,\tilde{p}_i)/\sigma_i^{uv}$. Thus there are at least k points $\tilde{p}_i$ such that $d_{\mathcal{M}}(p,\tilde{p}_i) < d_{\mathcal{M}}(p,P)$, which contradicts $P \in \mathcal{N}_{\mathcal{M}}$. Therefore, if $P \in \mathcal{N}_{\mathcal{M}}$, then $P \in \mathcal{N}^{uv}$.

Now consider if $P \notin \mathcal{N}_{\mathcal{M}}$ and $P \in \mathcal{N}^{uv}$. Thus there must be k data points $\tilde{p}_i \neq P$, $\tilde{p}_i \in \mathcal{N}_{\mathcal{M}}$, $d_{\mathcal{M}}(p,\tilde{p}_i) < d_{\mathcal{M}}(p,P)$. Since $P \in \mathcal{N}^{uv}$, P must be closer than at least one other $\tilde{p}_j$ to p in $\mathbb{X}^{uv}$. Therefore, since $\tilde{p}_j \in B$, $P \in B$. Since B is a constant diagonal matrix for all points in B, by Lemma 3, $d_{\mathbb{X}^{uv}}(p,P) = d_{\mathcal{M}}(p,P)\sigma_i^{uv}$ and $d_{\mathbb{X}^{uv}}(p,\tilde{p}_i) = d_{\mathcal{M}}(p,\tilde{p}_i)\sigma_i^{uv}$. Thus there are k points $\tilde{p}_i$ such that $d_{\mathbb{X}^{uv}}(p,\tilde{p}_i) < d_{\mathbb{X}^{uv}}(p,P)$, which contradicts $P \in \mathcal{N}^{uv}$. Therefore, if $P \in \mathcal{N}^{uv}$, then $P \in \mathcal{N}_{\mathcal{M}}$.

Therefore $\mathcal{N}_{\mathcal{M}}(p,k)$ and $\mathcal{N}^{uv}(p,k)$ have the same points, i.e. $\mathcal{N}_{\mathcal{M}}(p,k) = \mathcal{N}^{uv}(p,k)$.

4 Computation of MultiMAP

MultiMAP is outlined in Algorithm 1. For each data point, MultiMAP finds a set of nearest data points in each modality. The distances to these nearest neighbors are converted to a geodesic distances on a shared latent manifold by normalizing with respect to a radius value. The number of nearest neighbors and the radius depend on the data point and the modality of the neighbor. The nearest neighbor pairs and geodesic distances are used to construct a fuzzy

simplicial set and a MultiGraph. MultiMAP then initializes a layout of the data in a low-dimensional space to the spectral layout of the MultiGraph. MultiMAP proceeds to optimize the layout to minimize the weighted cross entropy between the fuzzy simplicial set and a fuzzy set of the data in the low-dimensional space. The optimization is performed using stochastic gradient descent. MultiMAP returns the optimized low-dimensional layout and the MultiGraph.

Algorithm 1: MultiMAP

function MultiMAP($\{\mathbf{X}^{uv}\}, \{I^{uv}\}, \{J^{uv}\}, S, \{\omega^v\}, k, d$)
  $N \leftarrow \sum_{u,v} |\mathbf{X}^{uv}|$ ▷ any input $\mathbf{X}$ can be replaced with D
  Initialize A to $\{\}$
  Initialize $\mu^{uv}_{i|j}$, $i \in I^{uv}$, $j \in J^{uv}$, to 0
  for all $(u, v) \in S$ **do**
    nn_query $\leftarrow \{\mathbf{x}^u_i \mid i \in I^{uv}\}$
    nn_reference $\leftarrow \{\mathbf{x}^v_j \mid j \in J^{uv}\}$
    nn_k $\leftarrow \lfloor k|J^{uv}|/N \rceil$
    $\{a^{uv}_{ij}\}, \{D^{uv}_{ij} \mid \exists a^{uv}_{ij}\} \leftarrow$ NearestNeighbors(nn_query, nn_reference, nn_k)
    $A \leftarrow A \cup \{a^{uv}_{ij}\}$
    for all $i \in I^{uv}$ **do**
      Binary search for σ^{uv}_i that satisfies Equation 1
      $\rho^u_i \leftarrow \min_{a^{uv}_{ij} \in A} D^{uv}_{ij} / \sigma^{uv}_i$
      for all $j \in \{l \mid a^{uv}_{il} \in A\}$ **do**
        $\mu^{uv}_{i|j} \leftarrow \exp(-\max(0, d_{\mathcal{M}}(\mathbf{x}^u_i, \mathbf{x}^v_j) - \rho^u_i))$
  for all $a^{uv}_{ij} \in A$ **do**
    $\mu(a^{uv}_{ij}) \leftarrow \mu^{uv}_{i|j} + \mu^{uv}_{j|i} - \mu^{uv}_{i|j}\mu^{uv}_{j|i}$
  MultiGraph $\leftarrow$ {nodes $\leftarrow \{\mathbf{x}^v_i\}$, edges $\leftarrow A$, weights $\leftarrow \mu$}
  $\{\mathbf{Y}^v\} \leftarrow$ InitializeEmbedding(N, d, MultiGraph)
  $\{\mathbf{Y}^v\} \leftarrow$ MinimizeCrossEntropy($\{\mathbf{Y}^v\}, A, \{\mu(a^{uv}_{ij})\}, \{\nu(a^{uv}_{ij})\}, \{\omega^v\}$)
  return $\{\mathbf{Y}^v\}$, MultiGraph

Nearest neighbor calculation is performed using the Nearest-Neighbor-Descent algorithm [14,60], which has an empirical complexity of $O(N^{1.14})$. Optimization of the low-dimensional layout using stochastic gradient descent closely follows [14,60,61]. The spectral layout of the MultiMAP tends to produce a good initial embedding, reducing the number of iterations needed for convergence. The runtime of the optimization scales with the number of edges in the fuzzy simplicial set, resulting in a complexity of $O(kN)$. Taken together, the complexity of MultiMAP is $O(N^{1.14})$. MultiMAP is highly efficient and readily scales to large datasets.

5 MultiGraph

In addition to a vector embedding of the data, MultiMAP produces a graph called the MultiGraph. The MultiGraph integrates the multimodal data into a

single graph structure. The MultiGraph is a neighbor graph of all data across all datasets on the shared manifold. Nodes that are connected by an edge share a neighborhood on the manifold. Edges with weights closer to 1 connect points that are closer on the manifold, and edges with weights closer to 0 connect points further away on the manifold. As described, the MultiGraph is not a k-NNG because nodes can have different degrees.

While the vector embedding of MultiMAP is useful for many analyses and visualization, we find that the MultiGraph can also be useful. The MultiGraph can be used for joint clustering, label transfer, link prediction, feature space imputation, and other analyses. Computing the MultiMAP is extremely efficient since there is no need to construct the low-dimensional fuzzy set or optimize the embedding.

6 Properties of MultiMAP

MultiMAP has several desirable properties that make it suitable for a wide variety of data and applications. We list these properties for convenience.

1. MultiMAP is highly efficient and readily scales to large datasets.

2. MultiMAP can be applied to any number of datasets simultaneously

3. MultiMAP is a nonlinear technique, allowing integration in settings with complex shifts and distortions.

4. MultiMAP can be applied to datasets with different feature spaces and dimensions. MultiMAP leverages features unique to particular datasets rather than operating only on features share by all datasets.

5. The influence of each dataset on the embedding of MultiMAP can be modulated by the user. This can be useful when a dataset is known to be of higher or lower quality.

6. In certain cases, we may desire that a subset of the data does not influence the integration. For example, consider if one dataset contains a population known not to be present in other datasets. We may desire the population to be included in the integrated embedding, but we would not want it to influence the layout of data from other datasets. MultiMAP can achieve this if the user eliminates D_{ij}^{uv} between points in this population and points in the other datasets.

7. MultiMAP can leverage side information, such as the class labels of data points, to integrate datasets in completely different feature spaces. Side information can used to calculate D_{ij}^{uv} and supplied to MultiMAP. In this case, the MultiMAP embedding will preserve the structure of each dataset independently while positioning them in the embedding so that points from the same class are near each other.

8. MultiMAP returns a MultiGraph which integrates multimodal data into a single graph structure. Construction of the MultiGraph is even more efficient than MultiMAP since the low-dimensional fuzzy set and embedding do not need to be constructed or optimized. The MultiGraph can be used with graph algorithms for clustering, node and link prediction, and other analyses.
