# Supplementary material for: MultiMAP: dimensionality reduction and integration of multimodal data
Source: Genome Biol. 2021 Dec 20;22:346. doi: 10.1186/s13059-021-02565-y (PMC8686224; doi:10.1186/s13059-021-02565-y)
Supplement: Supplementary file 3 — Additional file 3. Review history [file 13059_2021_2565_MOESM3_ESM.docx]

Review History

**First round of review**

**Reviewer 1**

**Were you able to assess all statistics in the manuscript, including the appropriateness of statistical tests used?**

Yes: Assessed

**Were you able to directly test the methods?**

Yes

**Comments to author:**

The paper describes a generalisation and extension of concepts that underpin UMAP. Specifically, the authors allow a Riemannian metric that enables geodesic distances to be calculated between points on the latent manifold given coordinates in ambient spaces (derived via PCA on the full and shared feature space) which are specific to the feature space of each dataset. This enables modalities of varying dimension and feature spaces to be handled. They also utilise an assumption, they name "invariance of similarity", that says data from different modalities which are close on the latent manifold, are also close in the coordinate systems of each dataset. The assumption of uniformly distributed data on the manifold, as in UMAP, then allows them to follow similar procedures to enable a final embedding. Notable differences include the availability of an underlying graph structure (referred to as the MultiGraph) and the use of weighted stochastic gradient descent to control the influence of each dataset when minimising the cross-entropy between the fuzzy set of the data on the manifold and its low-dimensional representation. The method benefits from being highly scalable due to the O(N^1.14) time complexity and this is demonstrated in the various experiments shown.

Methodologically the paper is interesting and this alone would be sufficient to merit publication in my opinion.

The authors have tested the methodology and others on a number of examples. As always in this area, it is difficult to interpret such results, due to the lack of ground truth. Evidence is based on anecdotal measures that are often limited by their reliance on previously published cell labels whose veracity (for the purposes of algorithmic benchmarking) is untested. Generally speaking, the speed of MultiMAP method is impressive and therefore, from a pragmatic perspective, it does not hurt just to "run it" to see what insights are gained from a dataset.

While the weighting parameters are highlighted as a "feature" of the methodology, they are also a device that introduces a substantial degree of subjectivity. It is not clear how such a weight could be set objectively and how sensitive results are too these values. All experiments appeared to use 0.8 for scRNA-seq and then 0.2 for all other -omics which ranged from ATAC, DropSeq and snmC-seq.

My main point for addition to the paper is a clear demonstration of the degree of sensitivity of outputs to these weightings. If the output is relatively robust to departures from the 0.8/0.2 combination then I would be satisfied but if output changes significantly I would be concerned about the utility of the outputs given dependence on these parameters.

**Reviewer 2**

**Were you able to assess all statistics in the manuscript, including the appropriateness of statistical tests used?**

Yes: No additional statistical review is recommended.

**Were you able to directly test the methods?**

Yes

**Comments to author:**

In this manuscript, Jain et al. present a computational framework, termed MultiMAP, for the dimensionality reduction and integration of single-cell multi-omic datasets. With the boosting scale of single-cell sequencing data, the accurate and efficient integration of single-cell multi-omic data is a computationally urgent task and should be of general interest to the community. The authors propose the MultiMAP method by generalizing the celebrated nonlinear dimensionality reduction method UMAP with the aim to place multi-omic data with distinct unmatched features in the same feature space. To achieve this goal, MultiMAP requires and unitizes the prior information of the shared feature space across modalities to link and couple the multi-omic data in the model.

The authors conducted comprehensive synthetic experiments, and single-cell multi-omic integration experiments. The authors demonstrated advantages of MultiMAP in performance over competing methods, e.g. Seurat 3, LIGER, conos and GLUER. Overall, this manuscript is very interesting, but I have some major concerns, as discussed below.

Major comments:

- MultiMAP's framework is built on the framework of UMAP, but it is never acknowledged as such in the main text.

- Although claimed to integrate single-cell multi-omic data with distinct unmatched features, it seems that MultiMAP did not fully solve this problem. In their implementation, MultiMAP resorts to unitize the prior information of the shared feature space across modalities, which was empirically pre-processed and obtained from the distinct unmatched features. For example, the gene activity scores of scATAC data, which were obtained by aggregating signals of multiple features in each gene body, were utilized by MultiMAP. MuliMAP was supervised by the information of the shared features while the compared methods, such as Seurat 3, were unsupervised, which makes the current comparisons unfair.

- What can be learned from or gained by incorporating the dataset-specific features (e.g., the cell type specific epigenetic information outside of gene bodies in the sc-ATAC data)?

- MultiMAP showed its effectiveness in correcting batch effects or modality differences caused by various experimental or technical noise on real biological datasets. Since it is the major improvement of MultiMAP, it deserves more explanations how MultiMAP solved this problem from the methodological perspectives. Is it due to that the shared feature space serves as the anchors to align the datasets? If so, it is not surprise to me that MuliMAP outperforms Seurat since Seurat learns purely its anchor points from data.

- Lines of important references, such as scAI (Genome Biol 21, 25 (2020). https://doi.org/10.1186/s13059-020-1932-8) ) and online iNMF (Nat Biotechnol 39, 1000-1007 (2021). https://doi.org/10.1038/s41587-021-00867-x ) are not cited. More importantly, there are no performance benchmarks with these methods.

Minor Comments:

- In the comparison, Seurat 3 was used. However, Seurat is up to v4 now.

- The discussion lacks a detailed comparison between integration methods, outlining the specific aspects in which MultiMAP might perform worse than other methods, and vice-versa. This makes it slightly harder for users to obtain a general conclusion from the study.

**Authors Response**

**Point-by-point responses to the reviewers’ comments:**

Reviewer 1

The paper describes a generalisation and extension of concepts that underpin UMAP. Specifically, the authors allow a Riemannian metric that enables geodesic distances to be calculated between points on the latent manifold given coordinates in ambient spaces (derived via PCA on the full and shared feature space) which are specific to the feature space of each dataset. This enables modalities of varying dimension and feature spaces to be handled. They also utilise an assumption, they name "invariance of similarity", that says data from different modalities which are close on the latent manifold, are also close in the coordinate systems of each dataset. The assumption of uniformly distributed data on the manifold, as in UMAP, then allows them to follow similar procedures to enable a final embedding. Notable differences include the availability of an underlying graph structure (referred to as the MultiGraph) and the use of weighted stochastic gradient descent to control the influence of each dataset when minimising the cross-entropy between the fuzzy set of the data on the manifold and its low-dimensional representation. The method benefits from being highly scalable due to the O(N^1.14) time complexity and this is demonstrated in the various experiments shown.

*Thank you greatly for your comment. We appreciate your enthusiasm about our algorithm.*

Methodologically the paper is interesting and this alone would be sufficient to merit publication in my opinion.

The authors have tested the methodology and others on a number of examples. As always in this area, it is difficult to interpret such results, due to the lack of ground truth. Evidence is based on anecdotal measures that are often limited by their reliance on previously published cell labels whose veracity (for the purposes of algorithmic benchmarking) is untested. Generally speaking, the speed of MultiMAP method is impressive and therefore, from a pragmatic perspective, it does not hurt just to "run it" to see what insights are gained from a dataset.

*Thank you very much for the feedback. To benchmark our algorithm against existing algorithms in the field, we use the following ground truth data: cell type labels generated by other members in the lab and by other publications as an independent and external source of validation.*

*In addition, we validate using a number of metrics -- including silhouette score, alignment score, Pearson correlation between distances in the high dimensional and low dimensional spaces -- which provide validation based on the structural qualities of the MultiMAP’s output and do not rely on ground truth labels. This provides an orthogonal and complementary form of validation to using ground truth labels.*

*In order to clarify this point, we have now modified the Benchmarking section in the Results section to include the following:*

*“To measure transfer accuracy and silhouette score we use cell type annotations generated by other publications or different members of our lab group as ground truth, to ensure an independent validation. Out alignment and structure preservation metrics are based on the structural qualities of MultiMAP’s output and do not rely on ground truth labels. This provides an orthogonal and complementary form of validation to using ground truth labels.”*

While the weighting parameters are highlighted as a "feature" of the methodology, they are also a device that introduces a substantial degree of subjectivity. It is not clear how such a weight could be set objectively and how sensitive results are too these values. All experiments appeared to use 0.8 for scRNA-seq and then 0.2 for all other -omics which ranged from ATAC, DropSeq and snmC-seq.

*Thank you very much for the advice. Throughout this paper we use a fixed default value of the weights at 0.8 for scRNA-seq and 0.2 for all other -omics, demonstrating that MultiMAP produces robust integration without the need to adjust the weighting. We intend the weighting parameter to be fixed at 0.8/0.2 for most circumstances. Having a fixed value for this parameter avoids subjectivity or over tuning that could be introduced by changing this parameter. This is very similar to the original UMAP algorithm, which has many parameters, e.g. min_dist, which could be changed to introduce subjectivity, but are instead held fixed and unchanged by the algorithm. Indeed, holding parameters fixed to avoid subjectivity is a common practice in algorithm design and similarly present in the Seurat, LIGER, Conos and GLUER pipelines.*

*In order to clarify this point, we have now included the following in the Methods and Results sections:*

*“Our results show that when integrating the MNIST data, for different choices of ωv, the datasets remain well integrated in the embedding space (Additional File S1a,b). For our real biological datasets, we use fixed default values of the weighting parameter (0.8 for scRNA-seq and 0.2 for all other -omics), demonstrating that MultiMAP produces robust integration without the need to adjust the weighting.”*

*“In addition, the integration produced by MultiMAP is robust to different choices of the weight parameters (Additional File S1c).”*

My main point for addition to the paper is a clear demonstration of the degree of sensitivity of outputs to these weightings. If the output is relatively robust to departures from the 0.8/0.2 combination then I would be satisfied but if output changes significantly I would be concerned about the utility of the outputs given dependence on these parameters.

*Nevertheless, even though we intend for the weighting parameter to be fixed at 0.8/0.2 for most circumstances, we demonstrate the effect of changing it in Additional File S1,a,b,c,d, using the MNIST and the mouse splenocyte scRNA-seq and scATAC-seq data. We see that while qualitatively the shape of the embedding changes (similar to adjusting the fixed parameters of UMAP), the desirable qualities of data alignment, cluster separation, and structural preservation remain essentially unchanged. This indicates a robustness of MultiMAP to changes in the weighting parameter.*

*In order to make this clearer in the text, we include the following sentences in the Methods and Results sections:*

*“Our results show that when integrating the MNIST data, for different choices of ωv, the datasets remain well integrated in the embedding space (Additional File S1a,b).*

*“In addition, the integration produced by MultiMAP is robust to different choices of the weight parameters (Additional File S1c).”*

*Which reference the following figure:*

*Additional File 1: Figure S1. MultiMAP's weight parameter. a. UMAP projections of the two halves of the MNIST handwritten digit images. b. MultiMAP embeddings as the weight parameters are varied. Each color is a different handwritten digit (0-9). When ω1 is larger than ω2, the embedding more closely resembles the projection of only X1; when ω2 is larger than ω1, the embedding more closely resembles the projection of only X2. For different choices of ωv, the datasets are well integrated in the embedding space. c. MultiMAP integration with varied weight parameters of published scATAC-seq15 and newly generated scRNA-seq data of the mouse spleen (n=1). d. Comparison of the MultiMAP integration of the spleen data as the weight parameter is varied -- in terms of transfer learning accuracy (“Transfer”), separation of cell type clusters as quantified by Silhouette coefficient (“Silhouette”), and preservation of high-dimensional structure as measured by the Pearson correlation between distances in the high- and low-dimensional spaces (“Structure”)*

*Thank you very much for your comments and suggestions, which have greatly improved our paper.*

Reviewer 2

In this manuscript, Jain et al. present a computational framework, termed MultiMAP, for the dimensionality reduction and integration of single-cell multi-omic datasets. With the boosting scale of single-cell sequencing data, the accurate and efficient integration of single-cell multi-omic data is a computationally urgent task and should be of general interest to the community. The authors propose the MultiMAP method by generalizing the celebrated nonlinear dimensionality reduction method UMAP with the aim to place multi-omic data with distinct unmatched features in the same feature space. To achieve this goal, MultiMAP requires and unitizes the prior information of the shared feature space across modalities to link and couple the multi-omic data in the model.

The authors conducted comprehensive synthetic experiments, and single-cell multi-omic integration experiments. The authors demonstrated advantages of MultiMAP in performance over competing methods, e.g. Seurat 3, LIGER, conos and GLUER. Overall, this manuscript is very interesting, but I have some major concerns, as discussed below.

*Thank you very much for your comments and suggestions. We are grateful that you appreciate the scalability and performance advantages of our approach.*

Major comments:

- MultiMAP's framework is built on the framework of UMAP, but it is never acknowledged as such in the main text.

*Thank you for pointing this out. We have now added mention and reference to UMAP throughout the paper:*

*In the Abstract:*

*“We introduce MultiMAP, a novel algorithm for dimensionality reduction and integration of multiple datasets, which generalizes the UMAP algorithm (McInnes et a!.) to the setting of multiple datasets with different dimensions. “*

*In the Introduction:*

*“MultiMAP generalizes the UMAP algorithm (McInnes et a!.) to the setting of multiple datasets with different dimensions, while implementing novel techniques for estimating the manifold, constructing a joint graph on the manifold, and optimizing the low-dimensional embedding to account for the limitations and the challenges of multimodal and multi-omic data.”*

*In the Methods:*

*“MultiMAP is based on a framework of Riemannian geometry and algebraic topology, and generalizes the UMAP framework (McInnes et a!.) to the setting of multiple datasets each with different dimensionality. ”*

- Although claimed to integrate single-cell multi-omic data with distinct unmatched features, it seems that MultiMAP did not fully solve this problem. In their implementation, MultiMAP resorts to unitize the prior information of the shared feature space across modalities, which was empirically pre-processed and obtained from the distinct unmatched features. For example, the gene activity scores of scATAC data, which were obtained by aggregating signals of multiple features in each gene body, were utilized by MultiMAP. MuliMAP was supervised by the information of the shared features while the compared methods, such as Seurat 3, were unsupervised, which makes the current comparisons unfair.

*Thank you very much for your helpful comment. MultiMAP incorporates unmatched features (a.k.a. dataset-specific features) into the integration. This is different from other methods including Seurat, Conos, LIGER, GLUER, which ignore and throw away unmatched features. We believe it is important to incorporate unmatched features so as not to throw away a substantial part of the data (sometimes unmatched features can be 99% of the data), and to avoid ignoring features that could reveal heterogeneity in cell type/state by capturing diverse facets of cell phenotype.*

*Importantly, we do not claim nor do we desire for our approach to be unsupervised and rely only on unmatched features. This is for two reasons: 1) If there are matched features that give clues to the alignment of data across omics, an ideal algorithm would leverage rather than ignore them. 2) In every multiomics setting we have encountered -- ATAC, RNA, methylation data, spatial data -- datasets always have some matched features.*

*In order to explain this clearly in the text, we have now added the following sentences to the Methods section:*

*“We note that MultiMAP leverages both shared and unshared features. We do not wish for our approach to rely only on unmatched features because correspondence between shared features provides valuable information across omics.”*

- What can be learned from or gained by incorporating the dataset-specific features (e.g., the cell type specific epigenetic information outside of gene bodies in the sc-ATAC data)?

*Thank you very much. This is an insightful line of questioning that highlights a key characteristic of our method that distinguishes it from other methods. Other methods like Seurat, Conos, LIGER, GLUER, integrate using only features shared between dataset. E.g. for scRNA and scATAC, other methods would ignore information in the ATAC outside the genes. In contrast, MultiMAP incorporates all features, including dataset-specific features, into the integration.*

*This is critical because:*

*1) The dataset-specific features often capture cell type or state heterogeneity not present in the shared features, e.g. cells may exhibit heterogeneity in chromatin structure outside of genes, or in the case of integrating scRNA with spatial data, heterogeneity in genes other than the very small number of ~1000 genes shared by scRNA with STARmap. Throwing away this eterogeneity could potentially stifle the discovery of new cell types or states.*

*2) Often, dataset-specific features are the majority of the data. In the case of integrating scRNA with STARmap, 99% of features are not shared when integrating with Seurat, Conos, LIGER, GLUER. This means a very large portion of the data is being thrown away when integrating with these methods potentially hindering analysis power and the discovery of rare cell types/states.*

*To demonstrate this, we showed through computational comparisons on the two datasets in Supplementary Figure 5 (included in the previous version of the manuscript) that MultiMAP benefits by incorporating the dataset-specific features. Using dataset-specific features results in qualitatively improved embedding and quantitatively, a significantly higher label transfer accuracy, alignment score, silhouette score, and structure preservation score.*

*Additional File 5: Figure S5. MultiMAP integration with all features vs. only shared features in the spleen scRNA-seq + scATACseq, and visual cortex STARmap + scRNAseq datasets. a. MultiMAP embeddings using all genes present in each dataset (intended use of MultiMAP). b. MultiMAP embeddings using only genes shared by all datasets in each integration. c. Comparison of the MultiMAP integration with all features vs. only shared features -- in terms of transfer learning accuracy (“Transfer”), separation of cell type clusters as quantified by Silhouette coefficient (“Silhouette”), mixing of different datasets as measured by fraction of nearest neighbours that belong to a different dataset (“Alignment”), and preservation of high-dimensional structure as measured by the Pearson orrelation between distances in the high- and low-dimensional spaces (“Structure”).*

*This shows that incorporation of non-shared features improves the separation of clusters which is useful for cell types clustering and separation.*

*To further demonstrate that using all features improves the integration, we compared the performance of MultiMAP with other integration methods on our most challenging data - the fetal thymus scRNA-seq and scATAC-seq data. We show that our method produces superior co-embedding and outperforms other integration methods. We added this comparison in Additional File 8: Figure S8.*

*Additional File 8: Figure S8. Fetal thymus scRNA-seq and scATAC-seq data. f) Embeddings returned by multi-omic integration methods on scRNA-seq and scATAC-seq datasets of the human fetal thymus*

*In addition, our integrated MultiGraph enables inference of joint developmental or differentiation trajectories across transcriptomics and chromatin accessibility data that allow the study of dynamic chromatin regulation alongside gene expression. Using non-shared features that account for the full epigenetic landscape of cells including distal enhancers, could help identify novel regulatory elements.*

*We appreciate your helping us to elaborate this point. We have now have added the thymus comparison in Additional File S8 and added this in the Discussion of the paper as follows:*

*“Existing integration methods require correspondence between all features profiled across omics technologies. In comparison, our method can incorporate different types of features, such as gene expression and open chromatin peaks or intergenic methylation, and thus takes advantage of the full power of multi-omics data. Ignoring the features unique to one dataset (as in most existing methods), may omit important information, for instance distinguishing features of certain subpopulations of cells and yield an integrated embedding that does not distinctly cluster all subpopulations. Dataset-specific features often capture cell type or state heterogeneity not present in the shared features, e.g. cells may exhibit heterogeneity in chromatin structure outside of genes, or in the case of integrating scRNA with spatial data, heterogeneity in genes other than the very small number of ~1000 genes shared by different technologies. Throwing away this heterogeneity could potentially stifle the discovery of new cell types or states. In addition, our integrated MultiGraph enables inference of joint developmental or differentiation trajectories that allow the study of dynamic chromatin regulation alongside gene expression. Using non-shared features that account for the full epigenetic landscape of cells,	including distal enhancers, could help identify novel regulatory elements.”*

- MultiMAP showed its effectiveness in correcting batch effects or modality differences caused by various experimental or technical noise on real biological datasets. Since it is the major improvement of MultiMAP, it deserves more explanations how MultiMAP solved this problem from the methodological perspectives. Is it due to that the shared feature space serves as the anchors to align the datasets? If so, it is not surprise to me that MuliMAP outperforms Seurat since Seurat learns purely its anchor points from data.

*Thank you very much for your comment. One of several critical distinctions between our algorithm and Seurat is that Seurat primarily uses a linear model, CCA, to correct for batch effects, while MultiMAP uses a nonlinear manifold-based approach. We believe the ability of our approach to correct nonlinear distortions, instead of only linear distortions like Seurat, makes it more suited to batch and technical differences between data which are unlikely to be purely linear. We demonstrate MultiMAP’s ability to correct nonlinear distortion in the paper using the Swiss Roll dataset.*

*In summary, the improved MultiMAP performance is likely not due to the lack of a need for anchors, but due to the lack of the assumption of a linear relationship between data sets. The non- linearity provides more flexibility and is more suited to the reality of biological variations across data modalities.*

*We have now added a sentence to articulate this difference in the Discussion section:*

*“Another limitation is that linear integration approaches such as CCA and NMF are not able to correct for nonlinear distortions between datasets. In contrast, MultiMAP uses a nonlinear manifold learning approach and we demonstrate that it can effectively integrate in the presence of nonlinear differences between datasets. We find this to be a significant advantage of MultiMAP both for multi-omic integration and batch correction.”*

- Lines of important references, such as scAI (Genome Biol 21, 25 (2020). https://doi.org/10.1186/s13059-020-1932-8) ) and online iNMF (Nat Biotechnol 39, 1000-1007 (2021). https://doi.org/10.1038/s41587-021-00867-x ) are not cited. More importantly, there are no performance benchmarks with these methods.

*Thank you very much for this suggestion. We have now included iNMF in the benchmarking analysis in Figure 5 and Additional File 6: Figure S6. Overall, iNMF performed quite similar to LIGER (with the exception of the run time that was faster), which is also based on NMF analysis. MultiMAP outperformed iNMF in terms of transfer accuracy, separation of cell type clusters, mixing of different datasets, preservation of high-dimensional structure, especially runtime -- where it takes orders of magnitude longer than MultiMAP and produced out-of-memory errors when run on 500,000 data points (218 GB RAM).*

*We could not make comparisons using scAI, because scAI integrates paired transcriptomics and epigenomics measurement from the same cell, whereas MultiMAP is designed for the much more common setting of unpaired multi-omics datasets measured in different cells.*

*We are grateful to you for directing us to this additional method and strengthening our paper.*

*Our new benchmarking results, including iNMF are shown in the following revised figures in our paper:*

*Figure 5. Benchmarking MultiMAP against existing approaches. a. Embeddings returned by multi-omic integration methods on different datasets. “X” indicates that the method terminated due to an out-of-memory error (218 GB RAM). b. Comparison of each method in terms of transfer learning accuracy (“Transfer”), separation of cell type clusters as quantified by Silhouette coefficient (“Silhouette”), mixing of different datasets as measured by fraction of nearest neighbours that belong to a different dataset (“Alignment”), preservation of high-dimensional structure as measured by the Pearson correlation between distances in the high- and low-dimensional spaces (“Structure”), and runtime. c. Wall-clock time of multi-omic integration methods on different sized datasets. Seurat and LIGER produced out-of-memory errors when run on 500,000 data points (218 GB RAM). To produce these datasets we subsampled the mouse primary cortex scRNA-seq and scATAC-seq data21 using geometric sketching34. The datasets were subsampled so that there are an equal number of cells in the scRNA-seq and scATAC-seq data until 100,000 cells. Since the scATAC-seq data had 81,196 cells in total, for the 500,000 cells comparison, we used an scRNA-seq of 418,804 cells. d. Comparison of capabilities and properties of each method. “Mapping” refers to the nature of the mapping employed by the method; “Max no. datasets” refers to the upper limit in terms of numbers of datasets accepted by the method; “Scalable to large data” refers to allowing a total of over 500,000 cells; “Data-set specific features” is whether the integration method allows information that is not shared across datasets; and “Dataset influence on integration” is whether the user can modulate the weighting of a given dataset relative to the others during the integration.*

*Additional File 6: Figure S6. Benchmarking MultiMAP using paired PBMC data. a. MultiMAP visualization of the Multiome RNA+ATAC PBMCs, colored by independently annotated cell type. b. Embeddings produced by alternative integration strategies, colored by omic technology. c. Comparison of each method in terms of transfer learning accuracy (“Transfer”), separation of cell type clusters as quantified by Silhouette coefficient (“Silhouette”), mixing of different datasets as measured by fraction of nearest neighbours that belong to a different dataset (“Alignment”), preservation of high-dimensional structure as measured by the Pearson correlation between distances in the high- and low-dimensional spaces (“Structure”), and runtime.*

Minor Comments:

- In the comparison, Seurat 3 was used. However, Seurat is up to v4 now.

*We thank the reviewer for this comment. When we started the development of our method, Seurat was on version 3. Fortunately, the updates from version 3 to version 4 are not relevant to our paper. The updates regard the processing of paired multi-omic datasets (different omics measured in the same cell), whereas our method and benchmarking and analysis is for the much more common situation of unpaired multi-omics datasets (different omics measured in different cells).*

*Just to be sure, we have now repeated the benchmarking analysis as shown below. The embedding and benchmarking of Seurat 4 for our data is essentially identical to before (except for slight variation in different runs due to random initialization).*

*Reviewer Figure 1. Comparison of Seurat v3 to v4. a) Embeddings generated from Seurat v3 of multi-omic data from mouse spleen and cortex, b) embeddings generated from Seurat v4 of multi-omic data from mouse spleen and cortex, c) Benchmarking of transfer learning accuracy (“Transfer”), separation of cell type clusters (“Silhouette”), mixing of different datasets (“Alignment”), preservation of high-dimensional structure (“Structure”), and runtime*

- The discussion lacks a detailed comparison between integration methods, outlining the specific aspects in which MultiMAP might perform worse than other methods, and vice-versa. This makes it slightly harder for users to obtain a general conclusion from the study.

*Thank you very much for pointing this out. We have now expanded the Discussion section to include a detailed comparison with other integration methods:*

*“Existing integration methods require correspondence between all features profiled across omics technologies. In comparison, our method can incorporate different types of features, such as gene expression and open chromatin peaks or intergenic methylation, and thus takes advantage of the full power of multi-omics data. Ignoring the features unique to one dataset (as in most existing methods), may omit important information, for instance distinguishing features of certain subpopulations of cells and yield an integrated embedding that does not distinctly cluster all subpopulations. Dataset-specific features often capture cell type or state heterogeneity not present in the shared features, e.g. cells may exhibit heterogeneity in chromatin structure outside of genes, or in the case of integrating scRNA with spatial data, heterogeneity in genes other than the very small number of ~1000 genes shared by different technologies. Throwing away this heterogeneity could potentially stifle the discovery of new cell types or states. In addition, our integrated MultiGraph enables inference of joint developmental or differentiation trajectories that allow the study of dynamic chromatin regulation alongside gene expression. Using non-shared features that include distal elements such as enhancers could help identify novel regulatory elements.*

*Another limitation is that linear integration approaches such as CCA and NMF are not able to correct for nonlinear distortions between datasets. In contrast, MultiMAP uses a nonlinear manifold learning approach and we demonstrate that it can effectively integrate in the presence of nonlinear differences between datasets. We find this to be a significant advantage of MultiMAP both for multi-omic integration and batch correction.*

*Crucially, some methods such as LIGER, Seurat, and other CCA or NMF approaches are challenged by scaling to large datasets because they require matrix factorization. In contrast, MultiMAP readily scales to hundreds of thousands of cells due to its graph-based algorithm.*

*An additional feature of MultiMAP, not present in other existing strategies, is that the influence of each dataset on the shared embedding can be modulated. This is useful when integrating datasets of different qualities, or when aligning a query dataset to a reference dataset. Comparison with existing methods for integration shows that MultiMAP outperforms or has close to best performance in every aspect investigated. MultiMAP is a robust and effective method for dimensionality reduction and integration of multimodal data, and is extremely fast and scalable to massive datasets.”*

**Second round of review**

**Reviewer 1**

The authors have adequately addressed my main concern about the weight parameters with no experimental results confirming that the method is generally robust to the choice of parameters.

**Reviewer 2**

All my concerns have been addressed.
